# A review: climate, contaminants and disease dynamics in lobster species *Homarus americanus, Homarus gammarus* and *Jasus edwardsii*

**DOI:** 10.3389/fphys.2026.1928932

**Published:** 2026-09-15

**Authors:** Noelle Babin, Amélie Guitard, Aleasha David, Carter Eagles, Benjamin deJourdan, Jae-Seong Lee, Michelle Theriault, Daniel Lane, Jeonghoon Han, Jordan Jun Chul Park

**Affiliations:** 1 Department of Science, Université Sainte-Anne, Church Point, NS, Canada; 2 Marine Research Centre, Université Sainte-Anne, Petit-de-Grat, NS, Canada; 3 Huntsman Marine Science Centre, St. Andrews, NB, Canada; 4 Department of Biological Science, College of Science, Sungkyunkwan University, Suwon, Republic of Korea; 5 Marine Biotechnology & Bioresource Research Department, Korea Institute of Ocean Science & Technology (KIOST), Busan, Republic of Korea

**Keywords:** climate change, disease ecology, lobster physiology, marine contaminants, multi-stressor interactions

## Abstract

Climate change, contaminant exposure, and emerging disease dynamics are increasingly recognized as interconnected stressors affecting marine crustaceans, including lobsters. This review compares three commercially and ecologically important species: *Homarus americanus*, *Homarus gammarus*, and *Jasus edwardsii.* Although these species differ taxonomically and ecologically, environmental stressors rarely act in isolation; climate change and contaminants interact to modify metabolic performance, molting, microbial dynamics and pathogen susceptibility. By synthesizing existing literature, this review identifies shared vulnerabilities and species-specific responses. Evidence suggests differences in thermal tolerance, developmental sensitivity and physiological plasticity, with early life stages consistently representing the most sensitive period across species. The American lobster has the strongest evidence linking warming waters to physiological stress and disease prevalence, particularly in Southern New England, whereas extensive research for European and Southern Rock lobsters remain limited. Across species, warming and ocean acidification often interact with contaminant exposure to alter immune function, molting, and recruitment. Key gaps remain in multi-stressor experiments, disease ecology and early life-stage research, emphasizing the need for integrative multi-stressor and cross-species frameworks to improve predictions of lobster resilience under accelerating environmental change.

## Graphical Abstract

**Figure:**
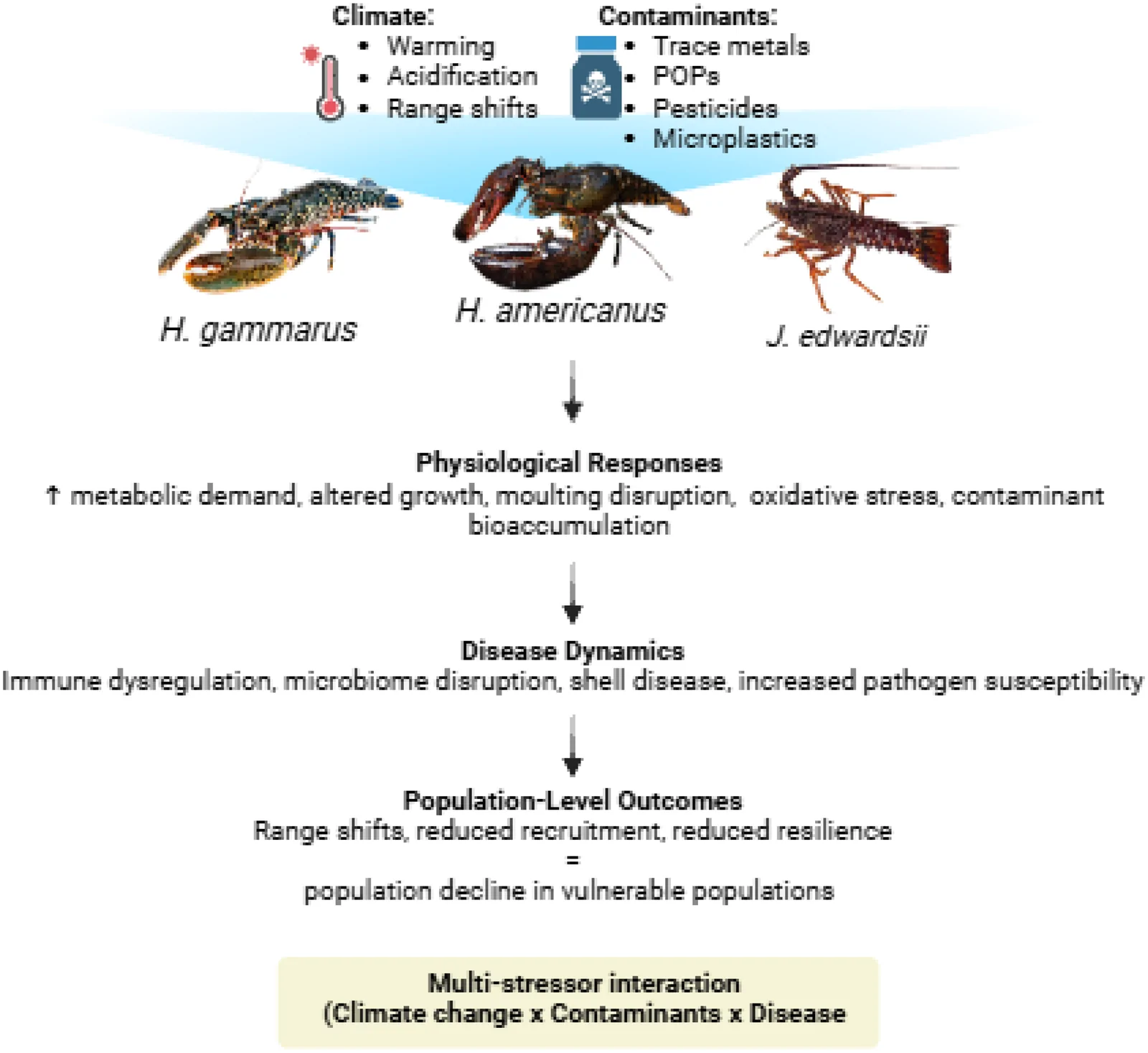

## 1 Introduction

Due to the fluctuating and often unpredictable nature of environmental stressors in marine ecosystems, organisms are frequently exposed to variable changes in temperature, chemistry and contaminant levels that can influence their physiology and survival. Among the affected species are lobsters, which represent the most economically valuable fisheries in North America with a combined value of over 3.6 billion dollars (CAD) in 2025, the most economically important species in Jersey’s (English Channel) commercial fishing industry valued at 4.7 million dollars (CAD) in 2024 and the top-earning single seafood export species in New Zealand valued at 286 million dollars (CAD) in 2021 (Binney et al., 2024; Fisheries and Oceans Canada, 2025; Maine Department of Marine Resources, 2026; Seafood New Zealand, 2022). This economic value makes lobsters a major driver of income for coastal communities and regional fisheries (Cheng et al., 2025). These fisheries anchor the economies of numerous coastal communities, whose resilience has been characterized across socioeconomic dimensions including business investment, financial health, and operational condition (Burnham et al., 2025).

In addition to their economic importance, lobsters play a key ecological role in benthic ecosystems. Lobsters act as keystone species of benthic ecosystems, influencing ecosystem structure, trophic dynamics and ecological functioning while also supporting economically important coastal fisheries worldwide (Eddy et al., 2014; Spanier et al., 2015). Ecologically, lobsters occupy multiple functional roles throughout development. Larval lobsters are pelagic and occupy a lower trophic position, feeding on planktonic organisms, whereas post-settlement juveniles and adults are increasingly benthic, preying on larger mid-trophic organisms, thereby functioning at a higher trophic level within coastal food webs (Phillips et al., 2013). As adults, lobsters are mid-trophic-level consumers, meaning their ecological role is to transfer energy from primary producers and primary consumers to apex predators (Phillips et al., 2013). This ontogenetic shift in habitat and feeding contributes to their role in structuring coastal food webs.

Beyond their economic and ecological roles, lobsters serve as valuable bioindicator species for marine environmental monitoring, owing to lobsters’ long lifespan, site fidelity and capacity to bioaccumulate contaminants. This has been demonstrated in the American Lobster, *Homarus americanus*, and the similar ecological traits imply that *Homarus gammarus* and *Jasus edwardsii* may show comparable potential (Chou et al., 2003; Turnbull et al., 2020).

Across their life stages, lobsters are exposed to multiple interactive stressors, including disease, climate variability and contaminant exposure, which can act both independently and in combination to affect survival and fitness. As the most important abiotic factor in marine ecosystems, climate change factors such as warming waters and ocean acidification affect lobster physiology (i.e. growth rates and maturity size), spatial distribution, reproductive processes, larval development, and disease susceptibility (Briones-Fourzán and Lozano-Álvarez, 2015). Similar climate-driven declines in physiological performance and increased susceptibility to disease have also been documented across other marine crustaceans such as crabs and shrimp (Behringer and Duermit-Moreau, 2021; Shields, 2019). Notably, many marine hotspots (e.g. south-east Australia, north-east Canada, Northern Indian Ocean, Galapagos/Eastern Tropical Pacific) are emerging or intensifying as a consequence of climate change, and these areas often coincide with regions where human populations rely heavily on marine resources (Hobday and Pecl, 2013).

Alongside their ecological and economic importance, lobsters also contribute to cultural backgrounds, especially associated with traditional livelihoods and coastal identities. Lobster fisheries are among the most economically valuable fisheries worldwide. North America reports the most lobster landings worldwide, reaching 50% of all landings with *H. americanus* and spiny lobsters, including *Jasus edwardsii*, reaching 23.6% of landings (Food and Agriculture Organization of the United Nations (FAO), 2024). On the other hand, global lobster landings from The United Kingdom of Great Britain and Northern Ireland reache about 10% (Food and Agriculture Organization of the United Nations (FAO), 2024). Their importance to coastal communities extends beyond economic value: in each region, harvesting is bound up with local identity and, in some cases, with long-standing systems of resource governance. Among Māori in New Zealand, harvest of *J. edwardsii* has for centuries been regulated by tribal customs restricting access by area, season and rotational cycle, an early form of spatial and temporal management, accompanied by ceremonial practice reflecting a spiritual relationship with the species (Spanier et al., 2015). In Norway, *H. gammarus* has been consumed in coastal communities since at least the 1500s and appears in 13th–14th century literature as a figure for the sea itself, indicating an association of comparable antiquity (Spanier et al., 2015). In Maine, the lobster fishery is associated with social and cultural identities of coastal communities. In fact, eight socioeconomic indicators have been identified for the resilience of lobster fisheries: business investments, coastal accessibility, community change, financial health, personal spending, physical and mental health, operational condition as well as risk taking (Burnham et al., 2025). This review focuses on three commercially and ecologically important lobster species: the American lobster (*Homarus americanus)*, widely harvested along the northwest Atlantic coast; the European lobster (*Homarus gammarus)*, which plays an important role in marine ecosystems; and the Southern Rock lobster (*Jasus edwardsii)*, a high-value species fished in Australasia, as illustrated in **Figure 1**.

**Figure 1:**
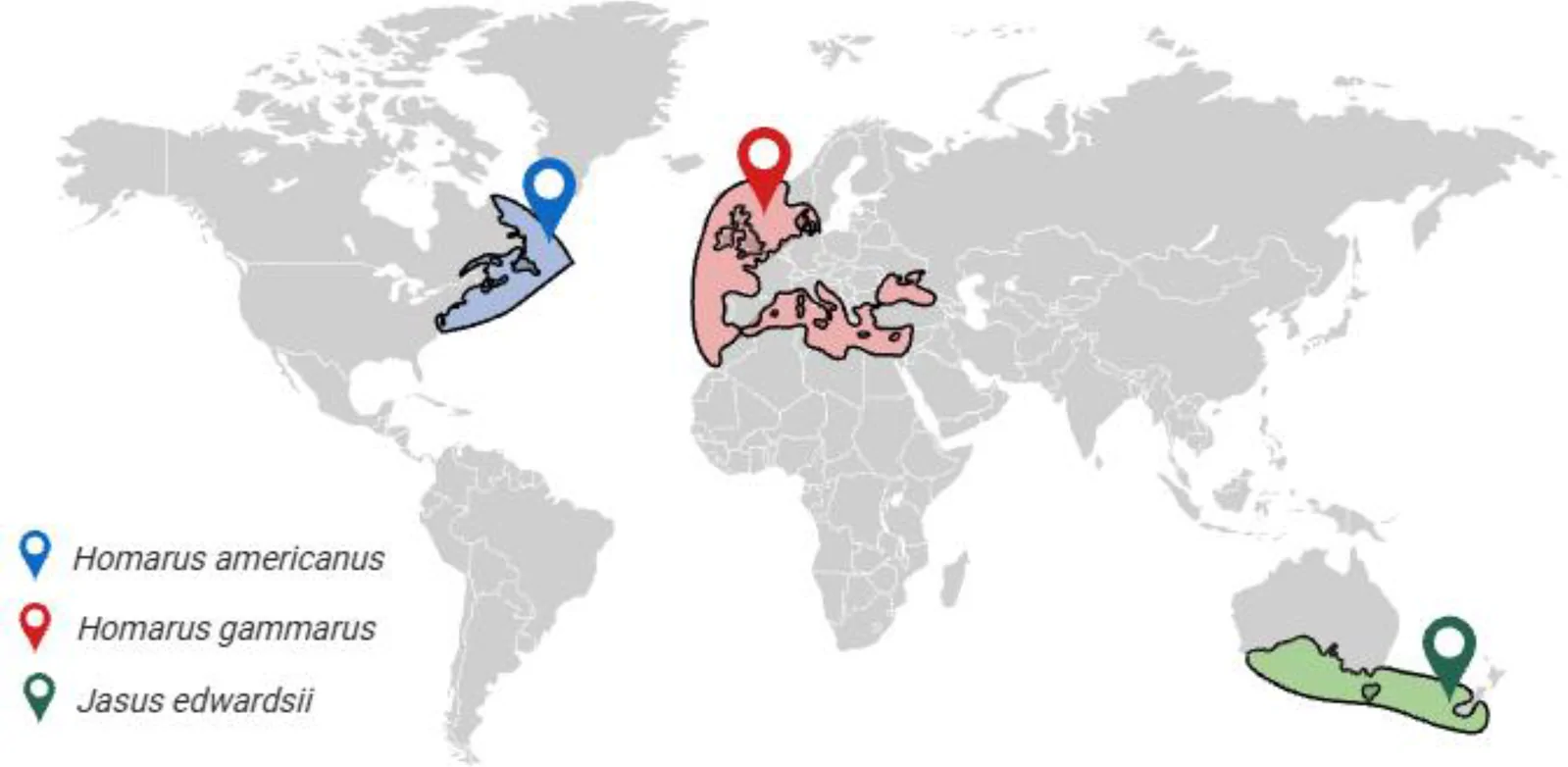
Geographical distribution of the three lobster species: *Homarus americanus, Homarus gammarus* and *Jasus edwardsii* (Bradford et al., 2015; Coleman et al., 2020*;* Gnanalingam et al., 2025; Goode and Bergman, 2024; Matić-Skoko et al., 2022; Pere et al., 2019; Phillips et al., 2017; Triantafyllidis et al., 2005).

*H.* americanus is distributed from Newfoundland and Labrador to North Carolina, where it inhabits rocky benthic habitats in the Northwest Atlantic (Atlantic States Marine Fisheries Commission, 2026; Fisheries and Oceans Canada, 2024). It supports the world’s most valuable lobster fishery, accounting for approximately 55% of global lobster supply (Food Export USA, ), making it a species of major economic importance. The life cycle of *H. americanus* spans several developmental stages, with a distinct duration and degree of exposure to environmental conditions. Eggs are carried by females for 10 to 12 months before hatching into planktonic larvae, which spend 3 to 10 weeks in the water column before moulting into a post-larval stage lobster (Fisheries and Oceans Canada, 2022). Following settlement, juveniles spend several years developing on the seafloor before reaching sexual maturity at 8 to 10 years of age, with a legal minimum harvest size of 82.5 mm carapace length (Fisheries and Oceans Canada, 2022). While reliable ageing methods remain limited, individuals are estimated to live up to 100 years in the wild (NOAA Fisheries, 2026). In terms of climate vulnerability, *H. americanus* undergoes seasonal moulting and reproductive cycles that can increase sensitivity to environmental stressors such as ocean warming and acidification (Atlantic States Marine Fisheries Commission, 2026; Fisheries and Oceans Canada, 2024; Groner et al., 2018). Warming seawater temperatures have been associated with shifts in moulting phenology and increased epizootic shell disease (ESD) prevalence, indicating that climate-driven changes can significantly influence the health and survival of *H. americanus* populations (Groner et al., 2018).

The European lobster (*H. gammarus*) is found along the European coastlines, where it occupies ecological niches similar to *H. americanus*, including rocky benthic habitats and seasonal moulting and reproduction patterns (Coleman et al., 2020). Economically, this species plays a relatively modest role in global lobster markets compared to the *H. americanus* and *J. edwardsii*. Despite this, it is frequently included in comparative lobster literature because it shares a genus with *H. americanus* (e.g., Davies et al., 2014a; Theberge et al., 2024). Genetic analyses suggest comparable levels of genetic variability and only a small but significant degree of divergence, indicating a relatively recent evolutionary separation and retained biological similarities (Hedgecock et al., 1977). The lifecycle of *H. gammarus* follows a broadly similar pattern to *H. americanus*, though with some important differences. Females typically reach reproductive maturity between 6 and 10 years of age (Marine Life Information Network (MarLIN), ). After eggs hatch, larvae spend 11 to 30 days in the water column before settling into the benthic juvenile stage, with reported life spans ranging from 11 to 20 years (Marine Life Information Network (MarLIN), ). The species is also subject to a minimum landing size of 88mm carapace length in UK waters (Shellfish FMP Policy Team, 2025). In terms of climate vulnerability, evidence from long-term benthic community changes and stocking experiments suggest that *H. gammarus* may exhibit relatively slow or uncertain recovery following environmental disturbance (Ellis et al., 2014; Franke and Gutow, 2004). Climate-driven shifts in community composition combined with limited understanding of post-release survival, recruitment and population-level effects, indicate constrained or poorly resolved recovery dynamics (Ellis et al., 2014; Franke and Gutow, 2004). This reduced capacity may increase vulnerability to chronic environmental change and limit population resilience following disturbances.

*J. edwardsii* is a clawless spiny lobster inhabiting temperate reef ecosystems in Southern Australia and New Zealand (Linnane et al., 2024; Phillips et al., 2013). It is considered one of the most economically important fisheries in New Zealand with approximately 392 million dollars (NZD) of exports in 2023 (Gnanalingam et al., 2025; Rock Lobster Industry Council, 2024). Its lifecycle begins with eggs attached to the female for 3 to 6 months, these eggs hatch into offshore larvae before transforming into post-larvae pueruli (Southern Rocklobster Limited, ). After settling from the water column, juveniles grow on the seafloor for 7 to 10 years before reaching sexual maturity, and adults can live up to at least 20 years (Southern Rocklobster Limited, ). Minimum legal-size limits are sex-dependent, set at approximately 60mm carapace length (Southern Rocklobster Limited, ). In terms of climate vulnerability, a key characteristic of this species is its exceptionally long larval phase (12 to 24 months) (Bradford et al., 2015). This prolonged pelagic duration increases exposure to changing oceanographic conditions, making recruitment and population connectivity particularly sensitive to climate-driven changes (Bradford et al., 2015). Consequently, because larvae remain in the pelagic for up to 24 months, and coastal waters are expected to undergo some of the most rapid climate driven changes, alterations in ocean circulation, and temperature may have disproportionately large impacts on larval transport, recruitment success and long-term fitness in this species (Linnane et al., 2024; Varela et al., 2023).

Collectively, these species span a broad range of geographic regions, habitats, thermal environments, and life-history strategies, including differences in larval duration, dispersal potential, and population connectivity. Accordingly, this review synthesizes current literature on climate change, contaminant exposure and disease dynamics in the American lobster (*H. americanus)*, the European lobster (*H. gammarus)* and the Southern Rock lobster (*J. edwardsii)* with the aim of identifying shared stress responses, species-specific vulnerabilities and major gaps in multi-stressor research.

## 2 Methods

Literature for this review was identified using Web of Science, PubMed, Google Scholar and the National Center for Biotechnology Information (NCBI) databases. Search terms included combinations of “*Homarus americanus*”, “*Homarus gammarus*”, “*Jasus edwardsii*”, “climate change”, “ocean warming”, “ocean acidification”, “contaminants”, “microplastics”, “disease”, and “microbiome”. Priority was given to most recent peer-reviewed studies examining physiological, ecological and diseases related responses to environmental stressors in lobsters (**Figure 2**).

**Figure 2:**
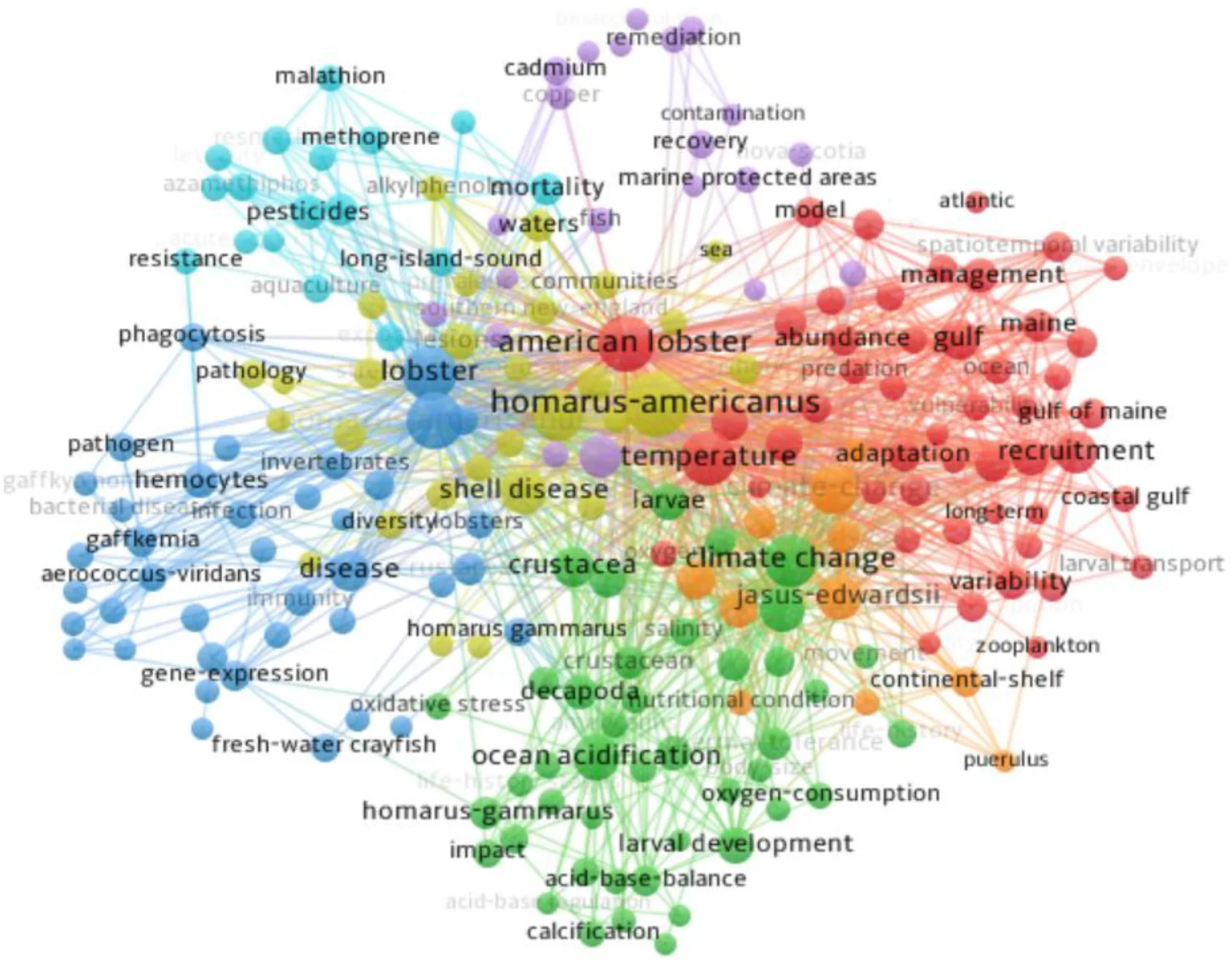
Bibliometric map of all keywords used to construct the co-occurrence network to capture both author defined and database-indexed terminology. Each node represents a keyword, with node size proportional to the frequency of occurrence across the analyzed articles. Node colors denote groups of closely related keywords that form thematic clusters, whereas the distance between nodes reflects the degree of relation, with shorter distances indicating stronger co-occurrence relationships. Source: Web of Science; visualization generated using VOSviewer; study period: 2000-2026.

## 3 Climate change

Climate change represents a multifaceted environmental stressor, incorporating but not limited to warming waters, ocean acidification and circulation changes, that alters physiological performance, species distributions, immune competence and disease susceptibility in marine crustaceans (Behringer and Duermit-Moreau, 2021; Khan et al., 2025; Monteiro et al., 2023; Shields, 2019). Lobsters are particularly vulnerable to climate related stress due to their complex larval phase and energetically demanding moult cycles (Amirah-Husna et al., 2026), which can be strongly influenced by changes in ocean temperature, acidification and circulation, while their benthic phase increases exposure to sediment-associated contaminants. Ocean warming, acidification, and changes in ocean circulation interfere with growth rates, reproduction, and survival probabilities of *H. gammarus, H. americanus* and *J. edwardsii* (Caputi et al., 2013; Leiva et al., 2022; Lopez-Anido et al., 2021). Although all three species are exposed to ocean warming, acidification, and variability in ocean circulation, species-specific differences in thermal tolerance, habitats, and developmental strategies result in distinct physiological and ecological responses to climate change. Importantly, climate-related stressors rarely act independently and may interact with contaminant exposure and disease processes to amplify physiological stress and reduce population resilience.

### 3.1 Ocean warming

Rising ocean temperatures represent a major climate-driven stressor in marine ecosystems, profoundly influencing the physiology, growth, and survival of lobster populations. Temperature directly affects metabolic rates, a key indicator of physiological performance and energy demand. As temperatures rise, metabolic costs increase, reshaping species’ energetic balance and influencing performance under future climate conditions (Shokri et al., 2026). For example, empirical studies on marine invertebrates in the Adriatic Sea suggest that warming can substantially increase standard metabolic rates, with projected energetic demand rising from 7.8% to over 27.3% by the end of the century, with stronger effects in cooler regions (Shokri et al., 2026). Climate models predict that sea surface temperatures along the Eastern Atlantic coast will increase by approximately 0.36 °C per decade between 2015 and 2050, nearly double the historical rate of warming (Varela et al., 2023). In the Northwest Atlantic, the American lobster (*H. americanus*) has been extensively studied in the Gulf of Maine, one of the fastest-warming marine regions globally, where sea surface temperatures are projected to increase by 0.86-2.89 °C by the end of the century (Fox-Kemper et al., 2021; Gulf of Maine Research Institute, 2018; Le Bris et al., 2018; Thomas et al., 2017). Consequently, the American lobster has become a key model for investigating the biological effects of ocean warming.

Thermal stress in ectotherms, like lobsters, typically unfolds in two stages: a cellular stress response followed by a decline in whole-organism performance (Kültz, 2020; Pörtner, 2002). Cellular stress response involves protective pathways (i.e. DNA damage response pathways, protein damage pathways, antioxidant enzyme pathways, cytoprotective metabolites pathways, transcriptional regulation pathways and post-transcriptional pathways), including regulation of heat shock proteins (*Hsp*s), that occur well before visible harm (Kültz, 2020). Once temperatures exceed the pejus temperature point (*Tp*), the growth, aerobic scope, and/or reproduction begin to suffer even though the organism is far from its lethal limit (Pörtner, 2002). The optimal temperature range for performance of life processes (e.g. moulting, survival) of *H. americanus* larvae ranges from 9 °C to 22 °C (Quinn, 2017). Interestingly, short-term exposure to elevated temperatures (i.e. 18 °C to 22 °C) can benefit growth rates, moulting and survival of larval *H. americanus* (Harrington et al., 2019; Lopez-Anido et al., 2021; Quinn, 2017). However, these benefits are offset by long-term trade-offs such as increased physiological stress represented by significantly higher hemocyte counts as well as an increase in differentially expressed genes associated with cellular stress response, ubiquitin proteins, Hsps, and DNA repair at 18 °C and 22 °C in post-larval (stage IV) lobster, consistent with an approach toward the pejus temperature (*Tp*), the threshold at which oxygen supply to tissues becomes limiting and physiological performance begins to deteriorate (Harrington et al., 2019; Lopez-Anido et al., 2021; Pörtner, 2002). Collectively, these findings indicate that 18 °C–22 °C likely constitutes the *Tp* range for *H. americanus* larvae, within which sustained exposure compromises physiological performance despite transient gains in growth and survival.

Larval stages of *H. americanus* exhibit stage-dependent thermal tolerances and experience ontogenetic shifts while their sensitivity to temperature varies across developmental stages, where stage IV is the most vulnerable to climate change (Annis et al., 2026). The aerobic scope (i.e. the difference between the maximum aerobic metabolic rate and the standard metabolic rate, both determined by measuring the rate of oxygen consumption) of stage I larvae is the most tolerable to cold and less tolerable to heat with a thermal range of 6 °C to 28 °C, stage II of 8 °C to 33 °C, stage III of 10 °C to 33 °C and stage IV of 9 °C to 27 °C before reaching critical temperatures (Annis et al., 2026). Additionally, stage I exhibited reduced survival under heat stress while stage II and III exhibited reduced survival under cold stress and reduced larval growth nearing critical temperatures for all developmental stages (Annis et al., 2026). When larvae (I – III) are exposed to high temperatures (25 °C) and low salinity (25 ppt), temperature appears to be the dominant factor by reducing survival, increasing heart rate and increasing the susceptibility to low salinity conditions (Guitard et al., 2026). Overall, lobster larvae experience sublethal effects of reduced growth rates outside of the optimal thermal range of 8 °C – 20 °C (Quinn, 2017). Collectively, these findings indicate that larval stages represent a major thermal bottleneck, with elevated temperatures accelerating metabolic demand and developmental processes while simultaneously increasing mortality risk. Temperature and oxygen tolerance in lobsters are interdependent, with thermal acclimation altering oxygen tolerance thresholds and oxygen conditions influencing thermal limits; while elevated temperatures also increase metabolic demand and accelerate energy depletion in larvae, potentially reducing physiological resilience under warming conditions (McLeese, 1956; Niemisto et al., 2025).

At higher temperatures of 16 °C and 18 °C, adult lobsters reach maturation at smaller sizes, producing lower quality eggs and hence larvae quality, which could reduce the fitness of *H. americanus* (Ascher et al., 2024). These temperatures are also associated with shifts in reproductive timing and altered larval survival dynamics (Ascher et al., 2024). For example, there has been a 31% decrease in fecundity of lobster from the Bay of Fundy between 2008 and 2013 which is likely due to the warming trends (Koopman et al., 2014). Recent studies demonstrate that adult American lobster thermal tolerance range is strongly linked to the lobster’s acclimation temperatures (Horricks et al., 2024). For example, it has been demonstrated that lobsters acclimated to cold water (5 °C) experienced euthanasia at 25.7 °C, while lobsters acclimated to warm water (15 °C) experienced euthanasia at 29.6 °C (Horricks et al., 2024). Lobsters acclimatized to ambient temperatures (~ 13.6 °C) had significant increases in *Hsp70, Hsp90, Hsc70* and *polyubiquitin mRNA* expressionwhereas lobster’s acclimatized to cold temperature (~ 0.4 °C) had no significant increases in gene expression when subjected to temperature increase of 13 °C (i.e. 26.6 °C and 13.4 °C), possibly suggesting higher basal-level gene expression potential to activate defense mechanisms including protein folding and repair functions of Hsps as well as targeting damaged proteins for degradation under thermal stress by polyubiquitin (Spees et al., 2002).

Collectively, these studies demonstrate that moderate warming can enhance larval growth and developmental rates in *H. americanus* within its optimal thermal range. However, these short-term benefits are consistently offset by increased metabolic demand, activation of cellular stress-response pathways (including heat shock proteins and polyubiquitin-related processes), and reduced reproductive performance and increased mortality under sustained exposure to elevated temperatures. In addition, strong stage-dependent differences in thermal tolerance indicate that larval stages represent a key physiological bottleneck, particularly near upper thermal limits where mortality risk increases. Consequently, ocean warming is likely to affect *H. americanus* populations primarily through reduced larval survival, altered recruitment success and declines in adult reproductive output, ultimately influencing population persistence under future warming scenarios. Comparable temperature-driven physiological responses are observed in *H. gammarus*, although available literature remains comparatively limited. The European Lobster (*H. gammarus*), inhabits various bodies of the ocean (East. Atlantic), such as the Mediterranean, Adriatic Sea and Black Sea, has a narrower thermal threshold than *H. americanus*. Notably, *H. gammarus* populations are exposed to markedly different baseline summer temperatures depending on region, with the Mediterranean (~24 °C) and Black Sea (21 °C-25 °C) historically reaching warmer summer temperatures than the North Atlantic (Krivoguz et al., 2021; Mercator Ocean International, 2025). Historically, *H. gammarus* in the North Atlantic has occupied waters ranging from 2 °C to 18 °C, considerably cooler than the Mediterranean and Black Sea conditions described above (Moland et al., 2011). This historical distinction may, however, be narrowing under climate change: in 2023, North Atlantic sea surface temperatures reached a record 25.3 °C, attributed to unusually strong solar heating combined with weaker-than-normal ocean cooling processes, superimposed on several preceding years of above-average warmth (Carton et al., 2025). These findings suggest that North Atlantic *H. gammarus* populations, historically adapted to cooler thermal regimes, may face increasing exposure to conditions more characteristic of the species’ warmer range; given the comparatively narrow historical thermal tolerance of these populations, the ecological consequences of such exposure remain uncertain.

Bottom water temperatures in the northern Adriatic Sea rose substantially between 2008–2012 and 2013–2017, by 1 °C–2 °C in winter and by as much as 2.5 °C in spring (Matić-Skoko et al., 2022). Over this same period, landings and catch per unit effort (CPUE) of European lobster increased significantly, a pattern potentially attributable to warming waters (Matić-Skoko et al., 2022). However, the combination of rising temperatures and increased abundance may create conditions conducive to overfishing (Matić-Skoko et al., 2022).

Much like *H.* americanus, the larval development of *H. gammarus* can benefit from warmer waters. There is an 80% increase in larval development at 22 °C (Schmalenbach and Franke, 2010). However, ocean warming may have even greater negative effects on the recruitment of *H. gammarus* (Leiva, 2023). For example, the metabolic rate of *H. gammarus* larvae experience stage specific thermal sensitivity, similar to *H. americanus*, under future ocean warming and acidification scenarios (Small et al., 2015). Oxygen consumption rates of stage I larvae were significantly lower while stage II, III and IV had significantly higher consumption rates when reared in high temperature waters (21 °C) compared to the control (17 °C) (Small et al., 2015). Stage I larvae also experienced decreased survival, while stages III and IV experienced an increase in survival at 21 °C (Small et al., 2015). These responses suggest that warming may initially accelerate growth development in *H. gammarus* larvae, but prolonged exposure to elevated temperatures creates energetic trade-offs that reduce long-term survival and recruitment. In addition, an increase in physiological stress responses, including the total phenoloxidase activity, which is associated with their innate immunity (Cerenius and Söderhäll, 2004), higher water content in muscle tissues and significant changes in the hepatopancreatic lipid contents have been associated with fasting condition of adult *H. gammarus* at a higher temperature of 12 °C (Albalat et al., 2019).

Collectively, these findings suggest that moderate warming can accelerate larval development and increase short-term performance in *H. gammarus.* However, these benefits appear to be offset by physiological stress, altered metabolic demands, and potential reductions in recruitment under prolonged exposure to elevated temperatures. It is important to note, however, that the aforementioned studies above were conducted on populations from different parts of the species’ range: Schmalenbach and Franke (2010); Small et al., 2015, and Albalat et al. (2019) used lobsters sourced from North Sea/North Atlantic waters, whereas Matić-Skoko et al. (2022) examined wild populations in the Adriatic Sea. Given the substantial baseline temperature differences between these regions described above, thermal responses observed in one population may not generalize to others across the species’ range. Overall, ocean warming may affect *H. gammarus* populations through changes in larval survival, recruitment dynamics and long-term population resilience, however, further studies are required to further elucidate the population-specific thermal thresholds and physiological mechanisms underlying these responses.

Much like the cold-water lobsters, *J. edwardsii* also experiences faster growth rates in moderately warmer temperatures (Phillips et al., 2017). Larvae reared at 18.2 °C had significantly larger body lengths in comparison to larvae reared at 10.5 °C and 21.5 °C (Bermudes and Ritar, 2008). In addition, the daily feed intake of stage I larvae significantly increased from 10.5 °C to 18.2 °C and 21.5 °C (Bermudes and Ritar, 2008). However, warm waters may also have negative effects on larvae. Almost all stage II individuals reared at 21.5 °C died due to starvation despite the presence of food, suggesting that warm waters are harmful and likely prevented feeding (Bermudes and Ritar, 2008). *J. edwardsii* has an optimal thermal range of 19 °C to 21 °C, with survival becoming significantly affected at 24 °C (Thomas et al., 2000). The optimal temperature for growth of post-puerulus J. *edwardsii* is a 20.6 °C and it decreased significantly at 24 °C while experiencing longer intermoult periods (Thomas et al., 2000). Lobsters held at 24 °C also experienced a significant decrease in oxygen consumption, suggesting respiratory stress under warming conditions (Thomas et al., 2000).

In comparison to the two *Homarus* species, which share a similar Atlantic habitat niche, adult *Jasus edwardsii* shows considerable metabolic plasticity in response to chronic warming: after at least 8 weeks of acclimation to projected 2070 future summer temperature of 21.5 °C (a 7.5 °C increase from the 14 °C annual average), standard metabolic rate declined by 29% relative to lobsters acclimated at colder temperatures, allowing them to conserve energy for non-maintenance activities such as feeding and moulting (Oellermann et al., 2022). This thermal benefit came with a trade-off, however, as the same warm acclimation reduced anaerobic escape capacity by up to 59%, potentially constraining repeated escape responses under sustained predation pressure (Oellermann et al., 2022). Despite this high thermal sensitivity of standard metabolic rate, warm-acclimated *J. edwardsii* (21.5 °C) also exhibited 10–20% lower maximum metabolic rates than other acclimation groups when tested at 14 °C (Oellermann et al., 2022). Marine heatwaves may be particularly disruptive because rapid temperature increases can exceed acclimation capacity, intensifying metabolic demand and immune dysregulation over short time periods. A study conducted on adult *J. edwardsii* from southern New Zealand demonstrated that under marine heatwave temperatures of 20-22 °C lobsters exhibited higher serum protein values, a presence of acyl carnitine metabolites and a significant increase in the maximum metabolic rate as well as the aerobic scope (Gnanalingam et al., 2025). Sub adult *J. edwardsii* acclimated to warm water temperatures (23 °C) do not display anti-predator responses that are observed at current water temperatures (20 °C) (Briceño et al., 2020). Overall, *J. edwardsii* demonstrates comparatively higher metabolic flexibility than the two *Homarus* species, allowing it to partially compensate for elevated temperatures through physiological adjustment. However, sensitivity during specific developmental stages, reduced performance near upper thermal thresholds and vulnerability to marine heatwaves indicate that warming may still affect recruitment success and long-term population resilience.

Although *H. americanus, H. gammarus*, and *J. edwardsii* differ in thermal tolerance, geographic distribution and habitat range, warming consistently affects metabolism, development, and survival across all three species, indicating a shared physiological sensitivity to rising ocean temperatures. However, the magnitude and expression of these responses vary, with the two *Homarus* species generally exhibiting more constrained thermal limits and clearer larval bottlenecks, whereas *J. edwardsii* demonstrates greater metabolic plasticity but remains vulnerable to rapid temperature increases and stage-specific stress, particularly under marine heatwave conditions. Furthermore, evidence from hatchery studies suggests that early-life environmental conditions can alter development, life-history traits and behavioral plasticity in *H. gammarus*, indicating that developmental environments may influence the capacity of juvenile lobsters to respond to future environmental change (Polverino et al., 2025). However, despite these species-specific differences, evidence consistently indicates that early life stages are the most sensitive to thermal stress across all three taxa. Consequently, future ocean warming is likely to influence population structure primarily through recruitment failure and downstream ecological effects rather than uniform adult mortality.

### 3.2 Ocean acidification

Relative to sessile calcifying invertebrates such as corals and mollusks, crustaceans are generally regarded as more resilient to ocean acidification, as many are strong iono- and osmoregulators able to compensate for hemolymph acidosis through effective acid–base regulation (Whiteley, 2011). Similar climate-driven declines in physiological performance, distribution and immune competence have not only been documented in lobsters but also across other marine crustaceans such as crabs and shrimp (Behringer and Duermit-Moreau, 2021; Monteiro et al., 2023; Shields, 2019). This resilience is not universal, however: sensitivity differs markedly among species and life stages, and poor regulators such as slow-moving, deep-sea and polar taxa remain vulnerable. Extrapolating resilience to lobsters therefore requires species- and stage-specific evidence.

Ocean acidification is a widespread consequence of increasing atmospheric CO_2_, which dissolves into seawater and lowers ocean pH while reducing the availability of carbonate ions essential for marine organisms (Turley and Gattuso, 2012). Since the Industrial Revolution, the average surface ocean pH has already decreased from approximately 8.16 to about 8.07, and is projected to decline further under future emissions scenarios, with estimates around 8.01 by 2100 even under mitigation pathways consistent with limiting warming to 2 °C (Kennedy, 2010). These changes alter marine chemistry and may affect a range of biological processes across marine ecosystems, particularly in organisms that rely on calcium carbonate structures such as corals, mollusks, and crustaceans (Turley and Gattuso, 2012). For calcifying species, reduced carbonate availability can impair shell and exoskeleton formation and increase the energetic cost of maintaining structural integrity (Keppel et al., 2012; Whiteley, 2011). In lobsters specifically, this process represents a chronic physiological stressor, particularly during moulting and juvenile development.

Studies on *H. americanus* indicate that early life stages are particularly sensitive to acidification (Lemmen et al., 2021). In fact, ocean acidification ha the most significant stage-specific impacts on juvenile American lobster’s body size, and cumulative effects across all life stages have the greatest impact on population (Tai et al., 2021). In addition, it can induce oxidative stress and alter acid-base regulation in embryos after only 24 hours of acute exposure, while larvae experience significantly shorter carapace lengths and longer development time under experimental acidification (pH = 7.7) and increased metabolic rates (Keppel et al., 2012; Sisti et al., 2025). While acute mortality may not always occur, sublethal effects (i.e. reduced growth, less frequent moults and deformities) can reduce long-term fitness (McLean et al., 2018). Interestingly, in *H. americanus*, sensitivity to elevated *p*CO_2_ varies across ontogeny, with larval stages (I-V) showing no significant effects on survival, development, or morphology despite strong metabolomic reprogramming, whereas juveniles exhibit reduced survival and delayed development under high acidification conditions (Noisette et al., 2021). This shift indicates that vulnerability to ocean acidification increases after metamorphosis, which represents a metabolic transition point in benthic-pelagic life cycles (Noisette et al., 2021). Notably, despite these sublethal effects on growth and development, calcification itself may not be compromised: adult *H. americanus* exposed to CO_2_ levels approximately 10 times pre-industrial concentrations for 60 days showed potential increases (i.e. net calcification levels ranging from 353 to 606.1), rather than reduced, exoskeletal calcification in contrast to the dissolution typically observed in non-regulating calcifiers such as mollusks (Ries et al., 2009).

Importantly, acidification may not act independently but instead may reduce resilience to concurrent stressors such as warming, contaminant exposure and disease. A 60-day laboratory study on subadult lobsters found that ocean acidification (pH = 7.6) and ocean warming of up to 25 °C significantly reduced hemolymph L-lactate concentrations and hemocyte counts were reduced by 61%, suggesting that these individuals are at risk of a compromised immune system potentially increasing susceptibility to pathogens (Harrington and Hamlin, 2019). Additionally, acidified conditions (pH = 7.6) narrowed the lobster’s thermal window by 1.1 °C due to reduced cardiac performance (Harrington and Hamlin, 2019). Recent transcriptomic evidence further suggests that combined ocean warming, and acidification alter the expression of genes involved in calcification, cuticle development, and immunity in early benthic juvenile *H. americanus*, with some immune-related genes showing trends toward down-regulation under multi-stressor conditions, potentially increasing susceptibility to injury and disease (San Antonio et al., 2025). Collectively, these findings indicate that ocean acidification affects *H. americanus* across multiple life stages, with early life-stages appearing particularly vulnerable due to reduced survival, delayed development and diminished physiological performance under both isolated and combined stressor conditions.

Similarly, early life stages of *H. gammarus* are the most vulnerable to ocean acidification. Laboratory studies demonstrate significantly reduced survival, shorter rostrum length in larvae experiencing high *p*CO_2_ conditions (~1160µatm) as well as significantly reduced superoxide dismutase activity (SOD), significantly reduced intermoult periods and reduced growth (i.e. body weight and carapace length) trends in larvae reared under future acidification scenarios (pH=7.8) (Leiva et al., 2022; Rato et al., 2017). Much like *H. americanus*, when ocean acidification is paired with warming waters it can limit the thermal window of *H. gammarus.* Larvae exposed to high *p*CO_2_ conditions (~1160µatm) and warm temperatures (24 °C) experienced additive effects on routine metabolic rates, survival and size which increased with temperature (Leiva et al., 2022). In contrast, adult *H. gammarus* appears more capable of compensating for acid-base disturbances: aerial exposure-induced hemolymph acidosis is fully compensated within this species, unlike in some other decapods (Taylor and Whiteley, 1989), suggesting that regulatory capacity may improve with life stage even as calcification related processes remain sensitive.

In addition, juvenile shell mineralization of *H. gammarus* is impacted under low alkalinity conditions (~ 830 μeq/L) by slowing post-moult calcification (Middlemiss et al., 2016). Juveniles experienced a reduction in bicarbonate uptake by 29% and a reduction in net base uptake by 22% during the 24-hour post-moult period (Middlemiss et al., 2016). However, additional experimental work is needed to better quantify physiological thresholds and long-term fitness consequences for this species under projected acidification scenarios. Collectively, these findings indicate that ocean acidification can impair growth, calcification, and physiological performance in *H. gammarus*, with larvae and juveniles appearing particularly sensitive to projected future conditions.

Although available evidence for *J. edwardsii* remains limited, ocean acidification may pose a particularly important risk during early development because of the species’ prolonged larval phase (Bradford et al., 2015). Near-future ocean acidification scenarios predict that *J. edwardsii* biomass under marine protection would only recover up to 58% of the biomass expected in the absence of acidification pressure (Cornwall and Eddy, 2014). Due to its magnesium-rich calcite exoskeleton, *J. edwardsii* may be particularly susceptible to dissolution and increased energetic costs associated with shell formation under acidified conditions, potentially experiencing similar vulnerabilities during post-moult mineralization to those observed in *H. gammarus* (Conroy, 2025; Middlemiss et al., 2016). Overall, *J. edwardsii* may be vulnerable to ocean acidification, particularly under conditions where multiple environmental stressors, including warming, act concurrently (Conroy, 2025; Coyle and Gnanalingam, 2026).

Long-term changes in reproductive structure have also been observed. Since 1969, a 20% decline in size at maturity has been observed in *J. edwardsii* populations in Southern New Zealand and fewer large but highly fecund females are expected today compared to past stock assessments (Coyle and Gnanalingam, 2026). This indicates a long-term shift in reproductive structure over time. The authors note that such changes are occurring in a context where marine fisheries are exposed to multiple environmental and anthropogenic stressors, including climate-driven changes such as ocean warming and acidification, alongside fishing pressure, which may collectively influence population dynamics (Coyle and Gnanalingam, 2026).

Collectively, studies across lobster species indicate that ocean acidification primarily induces sublethal physiological and developmental effects rather than immediate mortality, though responses may vary notably across life stages and physiological endpoints: growth, development, and immune function are frequently compromised, whereas calcification and adult acid-base regulation can remain robust or even enhanced, consistent with the broader pattern of regulatory resilience described for crustaceans generally (Whiteley, 2011). These effects are most pronounced during early developmental stages and following moulting, when energetic demands for growth and shell formation are highest. Across both *H. americanus* and *H. gammarus*, evidence also suggests that ocean acidification can interact with elevated temperatures, reducing physiological performance and narrowing thermal tolerance windows, thereby amplifying stress response under combined exposure. While *J. edwardsii* is less studied, available evidence may suggest similar vulnerability linked to its magnesium-rich exoskeleton and prolonged larval development, indicating that early life stages across all three species are likely to be the most sensitive to future ocean acidification.

### 3.3 Climate-driven distributional changes

Range shifts in species distributions provide clear signals of climate change consequences in lobster populations. *H. americanus* is experiencing a poleward and offshore range expansion in response to warming ocean temperatures, resulting in increased occupancy of previously cooler habitats (Greenhalgh, 2016). While northern populations have previously experienced biomass increases due to warming waters and expandable habitat suitability, such as in the Gulf of Maine, southern populations have experienced dramatic declines associated with thermal stress and disease outbreaks (Goode et al., 2019; Greenan et al., 2019; Greenhalgh, 2016; University of Maine, 2024). Due to warming waters, annual bottom temperatures are predicted to increase by 1.2 °C-2.4 °C by 2050, driving continued northward and offshore expansion away from southern nearshore regions (Rheuban et al., 2017). For example, in 2025, bottom temperatures in LFA 33 and LFA 34 increased by 2 °C compared to bottom temperatures of 2024 (Coldwater Lobster Association, 2025). In addition, increasingly warmer waters in the fall season have been linked to early spring egg hatching (Haarr et al., 2020). This pattern demonstrates thermal sensitivity combined with regional heterogeneity; warming is not universally beneficial but instead redistributes ecological risk. These changes are attributed to an increase in water temperatures, a decrease in salinity, a shift in oceanic currents, and predator redistribution (Cheng et al., 2025; Mason et al., 2024).

In contrast, *H. gammarus* has a limited capacity for dispersal (Triantafyllidis et al., 2005). Genetic differentiation of the European lobster using mitochondrial DNA revealed four major cluster groups: Mediterranean, northern Norway, Netherlands and Atlantic (Triantafyllidis et al., 2005). The abundance of lobster in the north-west region, as well as the reduced genetic diversities of north Arctic Circle populations, implies that sub-arctic lobster populations are geographically isolated (Jørstad et al., 2004). *H. gammarus* prefers low intertidal zones in the Atlantic Ocean, it is found at depths no greater than 50m (Holthuis, 1991). However, in Corsica, highest abundances have been recorded between 40m and 80m (Pere et al., 2019), indicating a deeper depth distribution in this region. The authors suggest this pattern may be related to elevated surface temperatures in the Mediterranean Sea, which can affect this cold-water species (Pere et al., 2019). Historically, this species has been subject to population decline, likely from overfishing, habitat destruction, and pollution (Franke and Gutow, 2004). To counteract population declines, many restocking programs and efforts have been established across Europe to either improve lobster redistribution or to rear market sized lobsters (Drengstig and Bergheim, 2013; Hinchcliffe et al., 2021). Nevertheless, the effectiveness of restocking programs remains inconclusive despite promising approaches (Ellis et al., 2014; Hinchcliffe et al., 2021). Unlike the American lobster, large-scale expansion has not been as clearly documented for *H. gammarus*, potentially due to geographic barriers, habitat discontinuities and slower recovery rates. Evidence suggests that recruitment variability in this species may be particularly sensitive to temperature anomalies and high *p*CO_2_ levels during the larval stages (Small et al., 2015). These climate-driven distributional shifts are occurring alongside intensive fisheries management focused on protecting mature females in both the Gulf of Maine and southern Norway; however, comparative modeling suggests that although such strategies can enhance egg production, their effectiveness is highly dependent on fishing intensity and species-specific life-history traits, therefore may be insufficient to prevent long-term declines under continued exploitation (Theberge et al., 2024).

Similar to *H. americanus, J. edwardsii* is also experiencing a latitudinal range shift. Climate-driven strengthening of the East Australian Current has shifted pueruli settlement patterns southward, indicating altered recruitment dynamics and potential redistribution of population biomass across the species’ latitudinal range (Pecl et al., 2009). This species has an exceptionally long larval phase, lasting from 12–24 months, during which larvae are transported by ocean currents (Bradford et al., 2015). As such, temperature-driven changes in current systems and productivity directly influence recruitment success (Caputi et al., 2013). Settlement of *J. edwardsii* exhibits large-scale regional synchrony influenced by climatic oscillations (e.g., El Niño-Southern Oscillation & Southern Annular Mode), while local environmental drivers such as wind strength and current transport direction contribute variably across sites, indicating that recruitment dynamics are governed by complex oceanographic processes rather than temperature alone (Hinojosa et al., 2016). Unlike the clawed lobsters, the extended larval phase of *J. edwardsii* amplifies climate sensitivity at early life stages, making recruitment highly climate dependent (Linnane et al., 2024). Pueruli recruitment is correlated with the East Australian Current, southward shifts in the current during the winter and spring result in reduced recruitment (Caputi et al., 2013). It is predicted that as water temperature increases in Tasmania, growth rates of *J. edwardsii* will also increase due to enhanced pueruli recruitment (Caputi et al., 2013).

Overall, range shifts in lobster populations provide strong evidence of climate driven redistribution, although the magnitude and direction of responses vary substantially among species. While *H. americanus* and *J. edwardsii* are showing clearer poleward or southward shifts linked to ocean warming and altered circulation patterns, *H. gammarus* appears more constrained in its capacity for rapid redistribution due to limited dispersal, habitat fragmentation and slower recovery dynamics. Collectively, these contrasting patterns highlight that climate change is not only reshaping lobster distribution but also exposing species-specific differences in dispersal and recruitment sensitivity that will ultimately determine future population resilience. Comparative responses to climate-related stressors across species are summarized in **Table 1**, with an integrated visual synthesis provided in **Figure 3**.

**Table 1:** Effects of climate stressors on lobster species.

Species	Life stage	Climate stressor	Key effects	References
*H. americanus*	Adult	Ocean Warming	Increased metabolic demand (↑ oxygen consumption, ↑ ammonia excretion), immune activation (↑ hemocyte counts), reduced reproductive performance (↓ egg quality, earlier maturation at smaller sizes), cardiovascular stress, up-regulation of heat shock and stress response genes (*Hsp 70, Hsp 90, Hsc70, polyubiquitin*)	(Ascher et al., 2024; Horricks et al., 2024; Klymasz-Swartz et al., 2019; Koopman et al., 2014; Spees et al., 2002)
*H. americanus*	Immature (Larval and Juvenile)	Ocean Acidification	Delayed development (↑ moult duration), reduced growth (↓ carapace length), increased metabolic stress, higher mortality, and reduced recruitment success	(Keppel et al., 2012; San Antonio et al., 2025)
*H. gammarus*	Larvae	Ocean Warming	Altered growth and development (↓ carapace length, ↓ development rate), increased oxygen consumption, elevated metabolic stress, and increased mortality	(Small et al., 2015; Leiva et al., 2022; Pere et al., 2019)
*H. gammarus*	Larvae	Ocean Acidification	Impaired calcification (underdeveloped rostrum, curled carapace deformities), increased immune activity (↑ phenoloxidase activity), reduced growth and increased mortality	(Agnalt et al., 2013; Leiva et al., 2022; Small et al., 2015)
*J. edwardsii*	Adults	Marine Heatwaves	Increased metabolic rate and oxygen consumption, elevated hemolymph protein concentrations, and altered metabolic scope	(Gnanalingam et al., 2025; Oellermann et al., 2022)

**Figure 3:**
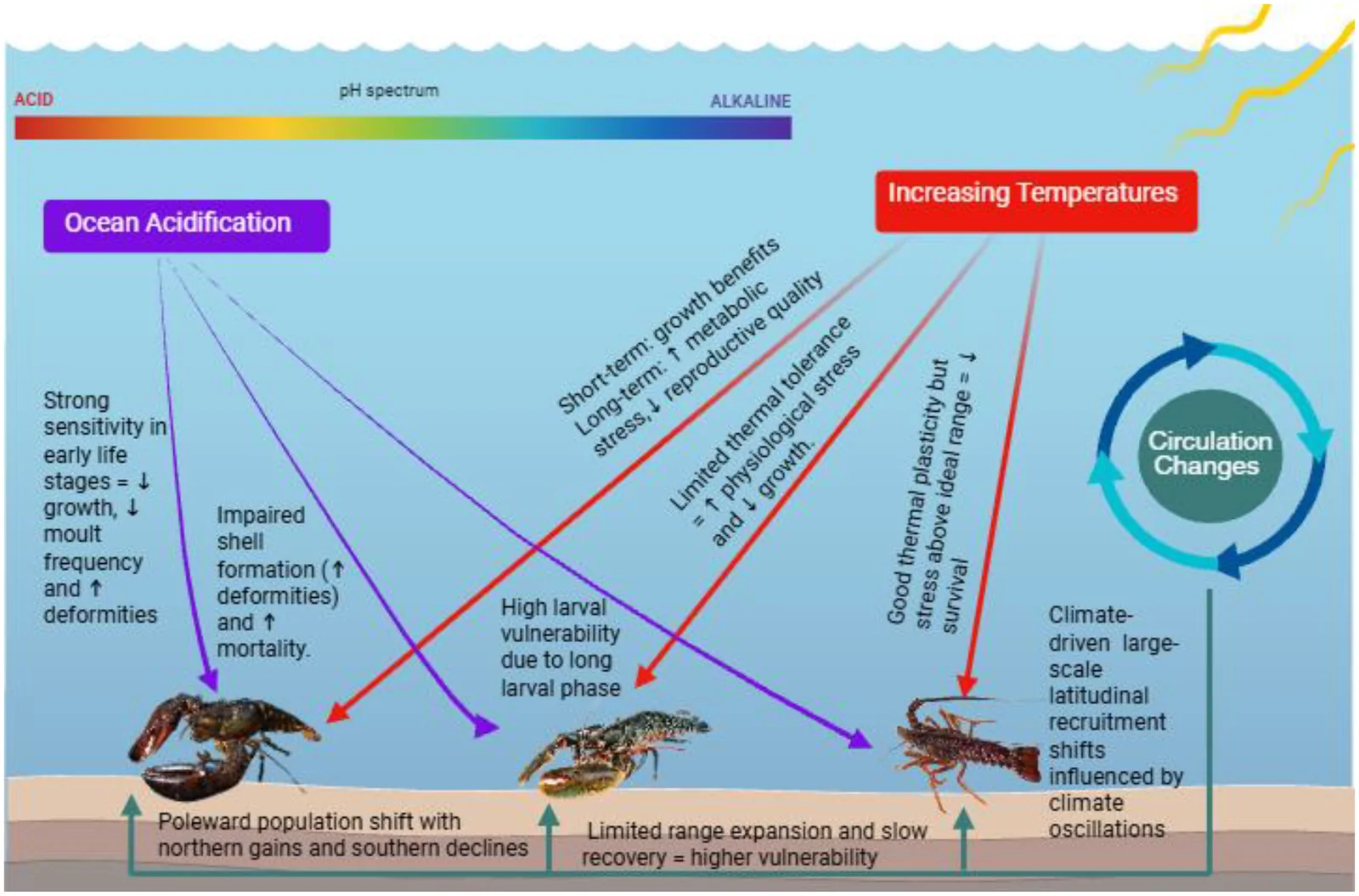
Species-specific responses of lobsters (*Homarus americanus, Homarus gammarus Jasus edwardsii*) to climate stressors (i.e. ocean warming, ocean acidification, circulation changes).

## 4 Contaminants

In addition to climate stressors, lobsters are also exposed to a wide variety of chemical contaminants that accumulate in sediment and benthic food webs. These chemicals not only act as stressors on their own but can also reduce the lobsters’ ability to tolerate other stressors thereby subjecting them to cumulative stressor effects (Segner et al., 2014). Due to their benthic lifestyle, long lifespan and trophic position as mid-level predators, lobsters are particularly susceptible to bioaccumulation of metals, persistent organic pollutants and emerging contaminants (Chen et al., 2023; Nikhil et al., 2026; Rao et al., 2025; Stoicov et al., 2025). Contaminant exposure often produces sublethal physiological effects in lobsters and occurs through sediment-associated pathways and trophic transfer (Pruell et al., 2000; Stoicov et al., 2025), as well as direct uptake across gill epithelia (Sá et al., 2009). When combined with climate stressors such as warming and acidification, contaminant exposure may amplify physiological stress and reduce overall resilience.

### 4.1 Trace metals

Tissue-specific accumulation patterns alone are of limited interpretive value; the toxicological relevance of trace metal exposure in lobsters ultimately rests on whether body burdens approach thresholds that induce oxidative stress or exceed detoxification capacity, and on the extent to which metal effects are modulated by co-occurring stressors such as temperature.

Trace metals are among the most studied contaminants in marine crustaceans due to their persistence and tendency to accumulate in sediments and tissues (Rao et al., 2025). In general, trace metals not only tend to accumulate in American lobster tissues but also alter hemolymph composition and oxygen-transport protein concentrations, particularly during moulting cycles (Chou et al., 2000; Engel et al., 2001). Indeed, recent field study from the Bay of Fundy and St Mary’s Bay has shown that metals can accumulate in hepatopancreas (i.e. As, Hg, Se, Pb, Cu, Co, Ni, Sr, Ca), muscle (i.e. Zn, Se, Fe) and shell tissues (i.e. Pb, Cu, Co, Be) of wild *H. americanus* (Mdaini et al., 2023). In addition to tissue-specific accumulation of trace metals, it has also been found to accumulate both in age/stage-specifically (e.g., juvenile and adult) as well as trace metal-specifically from the lobsters within Boat Harbour, Nova Scotia (Maltby, 2021). For example, there was no consistent correlation between the exposure duration and the metal concentration detected in adult lobsters, however, certain metals including aluminum (Al), iron (Fe), manganese (Mn), and strontium (Sr) have been found at a higher concentration in juveniles while cadmium (Cd), copper (Cu), mercury (Hg), silver (Ag), and zinc (Zn) were highly found in adult lobsters at levels above Canadian Food Inspection Agency (CFIA) guidelines (Maltby, 2021), The observed difference in trace metal accumulation across different life stages could be due to metabolic reorganization and hemolymph fluctuations (i.e. the concentrations of hemocyanin and its associated trace metals) during their moulting cycle, a stage at which they are physiologically sensitive and hence contribute to the differences in metal uptake and redistribution (Engel et al., 2001; Chou et al., 2003). As metals are known to induce oxidative stress in other crustaceans (e.g., *Palaemon macrodactylus, Procambarus clarkii*), several studies have shown differences in biomarker responses (Banaee et al., 2024; Zhang et al., 2021). However, no direct causal links between metal accumulation and biomarker changes were confirmed in *H. americanus* (Mdaini et al., 2023). Among trace metals, copper is particularly significant for lobster due to its tendency to accumulate in the hepatopancreas therefore affecting overall health (Chavez-Crooker et al., 2003). To deal with this, *H. americanus* has lysosomes that act as key detoxification sites to help regulate circulating copper levels and prevent copper toxicity (Chavez-Crooker et al., 2003). Nevertheless, it remains unclear whether detoxification mechanisms can adequately compensate for trace metal accumulation when lobsters are simultaneously exposed to other environmental stressors.

Research on trace metal accumulation in *H. gammarus* indicates that metals can impair core cellular processes, such as proteasome activity, findings in other decapods suggest comparable physiological responses to metal stress (Götze et al., 2014). A recent study determined that about 2-3% of European lobsters caught in Norway exceed maximum levels for mercury according to the Norwegian Food Safety Authority (NFSA) in muscle tissues (Nilsen et al., 2025). The uptake of mercury accumulates in the gills and the green gland of *H. gammarus* and can even lead to necrosis of the green gland (Brown et al., 1988). In addition, *H. gammarus* larvae can face lethal toxicity to mercury (LC_50_ = 48µg/L), copper (LC_50_ = 46µg/L) and cadmium (LC_50_ = 34µg/L) (Mariño-Balsa et al., 2000). An additional study, Devescovi and Lucu (2003), deduced that elevated levels of heavy metals resulted in an increase in hemolymph copper content. However, the hepatopancreatic tissues of *H. gammarus* have specialized transport mechanisms for copper, suggesting a detoxification role that influences metal homeostasis (Chavez-Crooker et al., 2003). Copper and cadmium exposure in *H. gammarus* has been associated with tissue redistribution and metal-binding responses consistent with detoxification processes (Devescovi and Lucu, 2003; Mariño-Balsa et al., 2000). In *H. gammarus*, the proteasome-mediated protein degradation activity was significantly suppressed by mercury (Hg), zinc (Zn), copper (Cu) and cadmium (Cd) (Götze et al., 2014). Limited data suggest that trace exposure may influence larval development and early juvenile survival (Mariño-Balsa et al., 2000), though comprehensive multi-life stage assessments remain scarce. Interestingly, *H*. *gammarus* has a high affinity for the radioactive metal technetium (Olsen and Vives i Batlle, 2003). Technetium accumulates the most in the hepatopancreas of male lobsters and the muscle tissue of female lobsters (Smith et al., 1998). There also appears to be sex differences among the lobsters’ gonads, ovaries accumulated 10–200 times more technetium than testes (Smith et al., 1998).

In *J. edwardsii*, trace metal research is primarily focused on identifying metal accumulation in coastal environments influenced by aquaculture (Turnbull et al., 2021), with fewer studies addressing controlled physiological responses to exposure. Trace metal concentrations, including arsenic, copper, cadmium, mercury, lead, selenium and zinc have been measured in the tissues of the Southern Rock lobster from coastal waters of Australia, demonstrating that wild populations accumulate many trace metals at quantifiable levels (Fabris et al., 2006). However, a study shows that juvenile *J. edwardsii* is capable of bio-regulating elevated levels of copper much like *H. americanus*, suggesting that this species may not be as susceptible to the bioaccumulation of copper (Graham, 2019). In contrast to copper, however, cadmium appears to be far less amenable to physiological regulation, with exposure linked to a range of adverse effects in other crustaceans such as reduced growth, impaired moulting, hormone disruption, altered microbial communities and oxidative stress, though species-specific responses in lobsters require further investigation (Das et al., 2019; Luo et al., 2015; Qian et al., 2024).

Across *H. americanus, H. gammarus* and *J. edwardsii*, trace metals are consistently shown to accumulate in key tissues and interfere with physiological processes such as moulting, immune function, and cellular homeostasis. However, most existing studies remain largely descriptive, focusing on documenting the presence and tissue distribution of metals rather than identifying mechanistic toxicity across life stages in addition to other stressors. This is particularly evident in the limited integration of sublethal physiological endpoints, multi-stressor interactions and long-term fitness consequences. As a result, there is a clear need for more comprehensive and comparative toxicological studies that move beyond metal detection to better quantify biological thresholds, functional impacts and species-specific sensitivity under realistic environmental exposure scenarios.

### 4.2 Emerging contaminants: persistent organic pollutants, pesticides and microplastics

Whereas acute toxicity dominates assessment of many contaminant classes, persistent organic pollutants (POPs), exert their principal effects through chronic low-dose exposure and bioaccumulation across trophic levels. Their toxicological significance is thus defined not by lethality thresholds, but rather by whether tissue burdens approach concentrations linked to sublethal physiological disruption.

POPs, including polycyclic aromatic hydrocarbons, polychlorinated biphenyls (PCBs), and other legacy industrial compounds, remain a significant environmental concern due to their persistence, toxicity, and ability to bioaccumulate and biomagnify within aquatic food webs. Even after regulatory restrictions and international bans, many of these contaminants remain in aquatic ecosystems due to their resilience (United Nations Environment Programme). PCBs are a group of man-made chlorinated organic chemicals that are particularly hazardous because they can persist in the environment for long periods and be transported over long distances through air, water, and migratory species, crossing international boundaries (United Nations Environment Programme). In coastal ecosystems, these compounds can persist in sediments and re-enter food chains, posing long-term risks to marine organisms and ecosystem health. In *H. americanus*, PCBs and other chlorinated organic contaminants have been detected in lobster tissues following exposure via contaminated sediment and prey in laboratory food-chain experiments, indicating that compounds can accumulate in lobster tissues through trophic transfer as well as sediment contact (Pruell et al., 2000). This bioaccumulation highlights the vulnerability of benthic species such as lobsters, which are closely associated with sediments where POPs often concentrate.

PCBs in particular pose a risk to *H.* americanus populations; PCBs (congeners 77 and 126) were 6 times more concentrated in juvenile lobster tissues (i.e. hepatopancreas and muscle) than initial sediment, suggesting that lobsters may be unable to metabolize this compound (Pruell et al., 2000). Field studies in the Outfall and Cape Cod Bay areas in Massachusetts, United States identified PCBs at concentrations exceeding the Environmental Protection Agency (EPA) screening-levels (Kane-Driscoll et al., 2008). Similar contamination has been documented in Canadian waters: Boat Harbour, Nova Scotia, was turned into an effluent discharge site and now contains sediments contaminated with organic compounds and polycyclic aromatic hydrocarbon (PAH) compounds, thus exposing *H. americanus* to these contaminants (Maltby, 2021). In the hepatopancreas tissues of these lobsters, two PAH congeners were identified, fluoranthene and pyrene, as well as all polychlorinated dioxins and dibenzofurans (PCDD/F) congeners (Maltby, 2021). Controlled toxicology studies show that exposure to petroleum-derived contaminants can induce physiological stress responses in adult *H. americanus* and reduce larval survival under experimental conditions (Berthod et al., 2021; Philibert et al., 2021). For instance, Berthod et al. (2021), demonstrated that exposure to petroleum products impairs hemocyte health and detoxification responses in adult *H. americanus*, with diluted bitumen causing particularly strong cellular impacts and incomplete recovery even after exposure ceased. PAH accumulation in eggs further suggested potential transgenerational effects of contaminant exposure (Berthod et al., 2021). Similarly, at earlier stages, exposure to oil-derived aromatic compounds, such as PAHs, caused rapid immobilization and mortality in stage I *H. americanus* larvae, with toxicity strongly associated with hydrophobicity and lipid accumulation potential (Philibert et al., 2021). These larvae also exhibited additive toxic effects across contaminant mixtures, highlighting the potential ecological risk posed by petroleum exposure during early developmental stages (Philibert et al., 2021). Beyond petroleum-derived compounds, per- and polyfluoroalkyl substances (PFAS) have also been detected in lobster tissues (0.37-5.15ng/g ww) and show bioaccumulation tendencies, with oxidative stress biomarkers (i.e. hydrogen peroxide and malondialdehyde) suggesting that PFAS exposure may induce physiological stress and weaken immune defenses, potentially increasing susceptibility to additional environmental stressors and diseases (Ogunbiyi et al., 2024).

Although direct studies of classic organic pollutants (e.g., PCBs, PAHs) in *H. gammarus* are scarce, crustaceans broadly have been shown to accumulate persistent organic pollutants and exhibit physiological and cellular responses, including altered metabolism and stress enzyme activity, when exposed to other contaminants (Camacho-Jiménez et al., 2023; Olsvik et al., 2015). Similarly, contaminant research on*J. edwardsii*is limited, particularly concerning POPs. However, a study demonstrates that *J. edwardsii* collected near the Astrolabe spill site exhibited significantly altered contaminant profiles, including elevated PAHs, arsenic and mercury concentrations, indicating site-specific contaminant accumulation following oil exposure (Ross et al., 2016).

In contrast to persistent organic pollutants, pesticide exposure in lobsters has been primarily investigated because of the active introduction of pesticides into lobster ecosystems through aquaculture use. Pesticides used to treat aquaculture farms, commonly to reduce arthropod pests or sea lice on salmon farms, often pose problems for non-target species such as *H. americanus*. Azamethiphos used in salmon aquaculture can cause immobilization, delayed mortality, and neurotoxic effects (i.e. reduced acetylcholinesterase activity) in larval *H. americanus* at environmentally relevant concentrations (as low as 2.9 µgL^-1^), while adult lobster appears more resistant but exhibit behavioral changes (de Jourdan et al., 2022). The sensitivity of adult female lobster to azamethiphos varies throughout the year (LC50 of 0.61 to 3.24 µg/L) being the most sensitive during spawning and moulting seasons when water temperatures are warmer (Burridge et al., 2005). Larval stages are therefore likely the most vulnerable to azamethiphos exposure (de Jourdan et al., 2022). Chlorpyrifos altered the expression of genes involved in stress responses (*glutathione peroxidase 3* and *heat shock protein 60*), hypoxia response (*hairy, astakine 2* and *hemocyanin*), moulting (*cytochrome p450 307a1* and *chitinase)* and immune function in stage IV *H. americanus* larvae after only 48 hours of exposure (Taylor et al., 2021). These changes suggest that chlorpyrifos can impair immune function, induce hypoxic stress, disrupt moulting processes and increase oxidative stress even at sublethal concentrations (Taylor et al., 2021). Pyrethroids have also been studied on *H. americanus.* Adult lobsters exposed to deltamethrin both chronically (10 days) and in pulses doses (every 15 or 30 minutes for 6 days) experience mortality and sublethal effects of immobility (Lyons et al., 2017). Lobsters exposed chronically had a lethal threshold of 14.7 ng·L^-1^ whereas lobsters exposed to high concentration (32 to 66 ng·L^-1^) pulse doses for 30 mins experienced mortality at all tested temperatures (i.e. 5 °C, 10 °C and 14 °C) and when exposed for 15 minutes mortality was greater than 50% (Lyons et al., 2017). When exposed to low concentrations of deltamethrin (8 to 13 ng·L^-1^) lobsters experience immobility and mortality (Lyons et al., 2017). Exposure to pyrethroid pesticides can cause sublethal developmental effects in juvenile *H. americanus*. Permethrin (100 µg/kg) reduces growth rates, decreases size increments and prolongs intermoult periods, indicating that pesticide contamination may impair development even in the absence of direct mortality (Daoud et al., 2023). Deltamethrin shows fewer measurable effects, although it causes a dose dependent increase in mortality with an LC50 of 2.5 µg (Daoud et al., 2023). Another common pesticide used on salmon farms, Emamectin Benzoate (EMB), has no effect on adult or stage I larvae of *H. americanus*; however, two batches of stage II larvae did not survive therefore reducing the proportion reaching stage IV from 6-7% to 0.5% (Asnicar et al., 2026). Surviving stage IV larvae exposed to EMB were also significantly smaller than control stage IV larvae (Asnicar et al., 2026). The combined effects of pesticides and other warming waters have also been studied in *H. americanus.* It is believed that one of the causes for the collapse of the American lobster population in western Long Island Sound (LIS) is due to the use of pesticides against mosquitos (Zulkosky et al., 2005). Stage I and II *H. americanus* larvae are very sensitive (LC_50_ 0.26-0.95µg/L^-1^ over a 48- and 96-hour period at 16 °C) to resmethrin, the active ingredient in the commercial product “Scourge” (Zulkosky et al., 2005). Malathion is less toxic to larvae than resmethrin with an LC_50_ of 3.7µg/L^-1^ whereas methroprene has no toxic effects of *H. americanus* larvae (Connecticut Agricultural Experiment Station (CAES) et al., 2014; Zulkosky et al., 2005). When combined, resmethrin or methroprene and warming temperatures (22 °C) have a significant effect on the phenoloxidase activity which had increased by 65% suggesting a significant effect of the immune system (Zulkosky et al., 2005). Despite the effects on young lobster, field sampling found no detectable levels of methoprene, resmethrin, bifenthrin, cyhalothrin or permethrin in the hepatopancreas or muscle tissues of adult *H. americanus* indicating that these contaminants may not bioaccumulate (Connecticut Agricultural Experiment Station (CAES) et al., 2014). Taken together, these studies indicate that pesticide exposure in *H. americanus* is highly compound-specific but consistently produces stronger effects in early life stages, with larvae showing the greatest sensitivity to mortality, developmental disruption and molecular-level stress responses. Across compounds, neurotoxicity, impaired moulting, and immune suppression emerge as recurring mechanisms while adults generally exhibit greater tolerance and more sublethal or behavioral effects. Although some compounds show limited evidence of bioaccumulation in adult tissues, short-term physiological and developmental impacts suggest that population-level consequences are likely driven primarily by early-life exposure rather than long-term accumulation.

Much like *H. americanus, H. gammarus* is a non-target species affected by azamethiphos and deltamethrin. Exposure to these pesticides was found to be acutely toxic to early larval stages (I and II) of *H. gammarus*, with immobilization and mortality occurring at concentrations below those used in salmon aquaculture treatments (Parsons et al., 2020). However, deltamethrin was identified as a greater environmental concern due to its high toxicity (EC_50_ of 0.6 and 0.4 ngL^-1^) and potential to disperse over large areas increasing the risk of exposure to wild lobster populations (Parsons et al., 2020). Juvenile *H. gammarus* exposed to the anti-parasitic teflubenzuron experience higher rates of mortality and deformities (Samuelsen et al., 2014). Lobsters were fed a medicated diet of 10mg/kg and 20mg/kg, both treatment groups experience a higher cumulative mortality than the control group however mortality did not differ significantly between both exposure groups and the half-life of teflubenzuron is 3.4 days (Samuelsen et al., 2014). In addition, up to 15% of treated lobsters experienced deformities (i.e. carapace, legs, cheliped, tail fan, abdomen and antenna) likely to hinder essential behaviors: foraging, feeding, respiration and movement (Samuelsen et al., 2014).

Similar to the other two species, *J. edwardsii* has become a non-target species affected by the usage of pesticides in aquaculture. Adult male lobsters exposed to 0.5 - 5µg/L^-1^ of azamethiphos experienced AChE inhibition and a decrease in muscle citrate synthase activity (Dounia et al., 2016). At the higher concentration (5µg/L^-1^), there was 93% cumulative mortality and significant changes (i.e. increased potassium and plasma protein, decreased chloride and sodium) to the hemolymph composition (Dounia et al., 2016). As an emerging contaminant class, the toxicological relevance of microplastics to lobsters is difficult to assess, both because realistic environmental concentrations remain poorly characterized and because microplastics may act indirectly, for example by altering the bioavailability of co-occurring contaminants, rather than through direct impact alone. Microplastics and nanoplastics, represent an increasingly recognized but poorly understood threat to lobster health, particularly through ingestion and trophic transfer. The ingestion of plastic materials by adult *H. americanus*, has been confirmed by the identification of rubber band fragments in lobster stomachs (Potocka et al., 2019). A recent study also documented the presence of microplastics in the muscle tissues of *H. americanus*, highlighting that even commercially harvested lobsters contain microplastics (LeBlanc et al., 2025). Experimental evidence from marine invertebrate, *Paracyclopina nana*, demonstrates that ingestion of nano-and microplastics (0.05-6µm polystyrene beads) can occur alongside egestion, but exposure is also associated with oxidative stress response and disruption of key physiological pathways, leading to sublethal effects on growth and reproductive performances; similar mechanisms may therefore also occur in lobsters, although species-specific responses remain poorly understood (Jeong et al., 2017). Microfibers, a dominant form of microplastic pollution originating primarily from synthetic textiles, represent an emerging contaminant that is of particular concern for lobster (Santonicola et al., 2023). Experimental studies have demonstrated that larval *Homarus americanus* readily ingest microfibers, which can become physically entangled within appendages or accumulate within the digestive tract, leading to reduced feeding efficiency, impaired energy allocation and decreased survival (Guitard et al., 2026; Woods et al., 2020). Microplastics may act as vectors for other contaminants, such as PCBs, PAHs, and heavy metals, by adsorbing and transporting these compounds, thereby altering their bioavailability and toxicity, thus exacerbating sublethal physiological stress (Arcadio et al., 2026; Conesa, 2022; Liu et al., 2021; Weis and Alava, 2023). However, ecological-scale impacts in lobsters remain unknown. Data on microplastic exposure in *H. gammarus* and *J. edwardsii* are limited, representing a key taxonomic research gap. Given similar benthic feeding and scavenging behaviors across lobster species, exposure risk is likely comparable to *H. americanus.*

Importantly, contaminant exposure rarely occurs independently of climate stressors. Elevated temperatures can increase metabolic rates, potentially accelerating contaminant uptake and altering detoxification efficiency, while ocean acidification may modify metal bioavailability and influence exposure pathways (Whiteley, 2011). Similar synergistic dynamics may exist in *H. gammarus* and *J. edwardsii*, though contaminant experiments are limited. Together, these interactions highlight a central theme of lobster ecotoxicology: environmental stressors act in combination rather than in isolation. However, despite clear evidence of interaction potential, empirical studies remain fragmented, underscoring the need for integrated experimental frameworks that assess combined contaminant and climate stressor effects across species and developmental stages. At the cellular level, this raises the possibility of interaction: exposure to trace metals has been associated with variability in oxidative stress biomarkers such as glutathione *S*-transferase and superoxide dismutase activities in *H.* americanus, although direct causal links remain unconfirmed, while PFAS exposure has been linked to elevated hydrogen peroxide and malondialdehyde levels suggestive of oxidative stress (Mdaini et al., 2023; Ogunbiyi et al., 2024). Independently, thermal stress in the same species has been shown to activate a broader cellular stress response, including the up-regulation of heat shock proteins and DNA repair mechanisms (Harrington et al., 2019; Lopez-Anido et al., 2021). Whether the cellular responses induced by these contaminants and temperature stress interact, act independently, or have a compounding effect, has not yet been directly tested in lobsters, representing a clear avenue for future multi-stressor research. The major contaminant types and their physiological effects across species are summarized in **Table 2**, while **Figure 4** provides a visual synthesis of contaminant bioaccumulation pathways and exposure routes in lobster.

**Table 2:** Effects of contaminants on lobster species.

Species	Contaminant type/stressor	Target tissue/system	Key effects	References
*H. americanus*	Heavy Metals (e.g., cadmium)	Hepatopancreas	Bioaccumulation and induction of oxidative stress responses (↑ GST, ↑ SOD), altered hemolymph composition, disruption of oxygen-transport proteins, age-dependent bioaccumulation patterns	(Chou et al., 2000; Engel et al., 2001; Maltby, 2021; Mdaini et al., 2023)
*H. americanus*	Microplastics	Digestive system	Physical obstruction and entanglement, reduced feeding efficiency, altered energy allocation (↓ oxygen consumption), increased mortality	(Guitard et al., 2026; Woods et al., 2020)
*H. gammarus*	Trace Metals (e.g., lead, cadmium)	Hemolymph	Impaired ion regulation and oxygen transport, suppression of proteasome activity, activation of detoxification pathways	(Devescovi and Lucu, 2003; Mariño-Balsa et al., 2000)
*J. edwardsii*	Trace Metals	Hepatopancreas	Bioaccumulation, oxidative stress, reduced growth, impaired moulting, decreased physiological performance	(Fabris et al., 2006; Turnbull et al., 2021)
*J. edwardsii*	Pesticides	Neurophysiological and metabolic systems	Neurotoxicity and physiological disruption (AChE inhibition and reduced citrate synthase), altered ion regulation, increased mortality	(Dounia et al., 2016)

**Figure 4:**
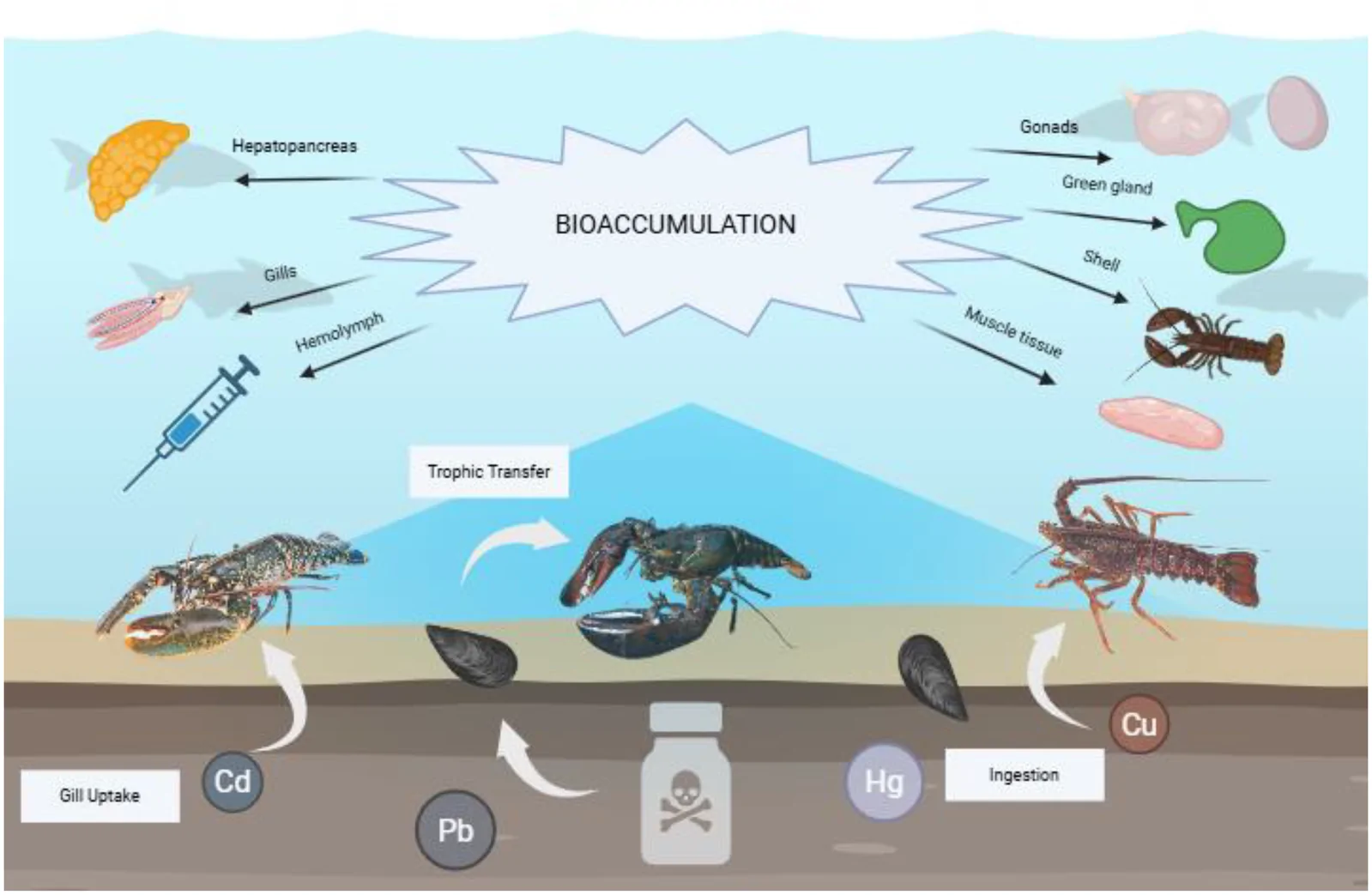
Bioaccumulation and trophic transfer of contaminants in marine ecosystems, particularly in lobster species. This schematic illustrates how contaminants (i.e. trace metals, POPs, pesticides, microplastics) enter marine food webs through environmental exposure pathways including gill uptake, ingestion, and sediment contact. These contaminants can then be transferred across trophic levels through predation, leading to progressive accumulation in higher organisms such as lobsters. Once absorbed, contaminants are distributed across multiple tissues, including the gills, hemolymph, hepatopancreas, muscle, gonads and green gland, where they may exert toxic effects.

## 5 Disease dynamics

Disease represents an important and increasingly studied outcome of environmental stress in lobster populations, particularly under conditions of rapid climate change and ocean warming (Rocker et al., 2025). Rather than functioning as isolated biological events, disease processes are better understood as the result of interacting environmental and biological factors that influence susceptibility and progression (Rocker et al., 2025). This perspective is particularly relevant for Epizootic Shell Disease (ESD), one of the most well-documented diseases in *H. americanus*, in part due to the visibility of its external lesions. As a result, ESD has been extensively studied compared to other lobster diseases, which may be simply less visible or harder to detect, rather than less prevalent. Nevertheless, ESD has been strongly associated with environmental degradation and changing ocean conditions in affected populations (Glenn and Pugh, 2006; Rocker et al., 2025).

### 5.1 Immunity

Lobster immune responses are primarily mediated by innate mechanisms, including circulating hemocytes and enzymatic pathways such as the phenoloxidase system, which plays a central role in pathogen defense and wound repair (Cerenius and Söderhäll, 2004). These immune processes are highly sensitive to environmental conditions and closely linked to metabolic status. For example, the Indian spiny lobster, *Panulirus homarus*, has a significant reduction in total hemocyte count and phenoloxidase activity when exposed to acidic/alkaline conditions, hypoxia and elevated ammonia-N concentrations (Verghese et al., 2007). These lobsters also experience significant reductions in total hemocyte count in both high and low salinity environments (Verghese et al., 2007). In *H. americanus*, elevated temperatures have been shown to increase hemocyte abundance and alter hemolymph chemistry, indicating activation of stress-related immune pathways (Harrington et al., 2019). Chronic activation of immune pathways during prolonged pathogen exposure can lead to immune dysregulation and reduced efficacy of defense mechanisms, as seen in experimental infections with *Anophryoides haemophila*, where extended immune engagement compromised host hemocyte profiles and defense enzyme activity (Fazelan et al., 2026). Similarly, in *H. gammarus*, increased phenoloxidase activity under elevated temperatures and nutritional stress suggests heightened immune activation, though this response is energetically costly and may not be sustainable under chronic stress conditions (Albalat et al., 2019). *J. edwardsii* shares the same type of innate immune system as *H. americanus* and *H. gammarus*; it is often characterised by two parameters: total hemocyte count and phenoloxidase activity (Zha et al., 2017). In general, elevated phenoloxidase activity is considered indicative of immune activation following pathogen exposure, while declining hemocyte counts are commonly linked to stress or infection (Alimin et al., 2024). An increase in these parameters can therefore reflect an individual actively responding to an infection rather than a compromised immune state; however, if elevated activity persists over time, it may instead signal a shift toward dysregulation, where sustained demand outpaces the lobster’s capacity to maintain effective defense. Distinguishing between these possibilities is often difficult without additional physiological and pathogen-load data, and this ambiguity should be considered when interpreting immune biomarkers in lobster stress studies. Overall, available evidence indicates that lobster immune responses are highly sensitive to environmental conditions, with stressors altering hemocyte abundance and phenoloxidase activity across species. Because these immune responses are energetically costly, prolonged environmental stress may reduce immune efficiency and increase susceptibility to disease.

### 5.2 Disease and lobster microbiome

Disruptions to immune function can influence interactions between the host and its associated microbial communities (Liu et al., 2023), potentially increasing susceptibility to opportunistic infections under environmental stress. In natural populations, disease may also be under-detected, as many infections are sublethal, internally expressed or transient, and therefore not consistently captured in field monitoring or linked to observable outbreaks. Lobsters are susceptible to a range of bacterial, viral and parasitic infections, with shell disease representing one of the most well-documented conditions in clawed lobsters. Baseline microbiome studies in Atlantic Canada show that the shell surface of apparently healthy lobsters is dominated by diverse bacterial groups, including *Gammaproteobacteria, Alphaproteobacteria, Flavobacteriia, Verrucomicrobiae, Saprospiria, Acidimicrobiia*, and *Planctomycetia*, with *Flavobacteriia* being previously associated with epizootic shell disease (ESD) also present (Koepper et al., 2023). Epizootic shell disease in *H. americanus* is characterized by cuticular lesions associated with complex microbial communities, with lesions progressing under elevated temperatures and disrupted microbiome dynamics (Kim et al., 2025; Tlusty et al., 2007). Recent research suggests that ESD and impoundment shell disease are not caused by a single pathogen but are instead linked to microbial dysbiosis, where environmental stressors disrupt the natural microbial community on the lobster’s shell, allowing opportunistic bacteria to proliferate (Argenta and Clark, 2025; Meres et al., 2012). Field studies have shown that lobsters exhibiting shell disease can have significantly higher concentrations of several trace metals, including mercury, copper, arsenic, calcium, zinc and cadmium, in hepatopancreas and other tissues compared with healthy individuals, suggesting a possible synergistic effect between contaminants and disease susceptibility (Vassiliev et al., 2005*).* In Southern New England, elevated temperatures and environmental stressors have been associated with increased prevalence and severity of ESD. Experimental research indicates that elevated temperatures can alter hemocyte abundance and physiological stress markers in *H. americanus* (Harrington et al., 2019). ESD is not limited to external shell damage but is associated with systemic physiological disruption. Transcriptomic evidence shows differential expressions of *anti-lipopolysaccharide factor (ALF*) gene increased across multiple internal tissues, including the hepatopancreas, intestines, green gland and testis, indicating that infection can impair immune function, energy metabolism and stress responses beyond the cuticle (Kim et al., 2025). The hepatopancreas also exhibited decrease expression of *hemocyanin* genes and a decrease in the expression of *heat shock* proteins in the green gland (Kim et al., 2025). Prevalence of ESD has been highest in regions experiencing sustained warming trends, suggesting that disease may serve as a biological indicator of environmental degradation (Rocker et al., 2025). Additionally, juveniles inhabiting low pH environments are more susceptible to shell disease (McLean et al., 2018). Associations between contaminant burden and crustacean shell disease prevalence have been proposed, however it has yet to be confirmed (Leblanc and Prince, 2012). On the contrary, laboratory studies demonstrate that embryos and larvae of *H. americanus* do not appear to be affected by environmental conditions, instead bacterial communities are determined by life history stages, in which they share only 3 core members: *Rubritalea, Delftia* and *Stenotrophomonas* (Koshak et al., 2026). While direct causation remains complex and multifactorial, the convergence of warming and contaminant burden provides strong support for a multi-stressor disease framework. Other disease agents, such as gaffkemia, which is caused by *Aerococcus viridans*, and parasitic infections, have also been repeatedly documented in *H. americanus* and are often associated with handling stress and high-density conditions (Clark et al., 2016; Handlinger et al., 2006). *Anophryoides haemophila* is a lethal protist that causes lesions that are first observed in gills and connective tissue (Athanassopoulou et al., 2004). A study analyzing expression changes in hepatopancreas genes of lobster subject to *A. haemophila* revealed 152 immune and stress related genes (Fazelan et al., 2026). Among those key immune-related genes, *anti-lipopolysaccharide factors (ALFs), pentraxin serum amyloid A (SAA)* and *lectins*, were differentially expressed, demonstrating the complexity of a lobster’s immune system (Fazelan et al., 2026). The differential expression of *ALFs* (i.e. up-regulation of *HA-ALF-L1, HA-ALF-*L2 and *HA-ALF-L1-*like as well as a down-regulation in *HA-ALF-L6-like-b* and *HA-ALF-*L6) is distinct from those reported in the cases of other pathogens indicating that *H. americanus* can utilize pathogen-tailored antimicrobial responses (Fazelan et al., 2026). This represents a shift from a traditional pathogen-centered view of disease toward a more integrative framework in which host condition and microbial community structure are equally important.

Comparatively, disease dynamics in *H. gammarus* are less extensively studied, although shell disease and parasitic infections have been observed in European lobster populations large-scale epizootic outbreaks comparable to those in Southern New England have not been widely described, which may reflect differences in environments as well as a lack of long-term monitoring (Ellis et al., 2014; Davies et al., 2014a; Small et al., 2015). Evidence from marine conservation zones suggests that no-take areas can increase *H. gammarus* density and size but may also coincide with higher prevalence of shell disease and injury, particularly in larger individuals (Davies et al., 2014a). While pathogen screening indicates low occurrence of major infectious agents, these findings highlight that spatial protection can produce complex and potentially trade-off driven outcomes for lobster health, reinforcing the need for long-term, pre- and post-establishment monitoring in Marine Conservation Zones (Davies et al., 2014b). *Microbiome* research in *H. gammarus* has shown that gut bacterial communities vary with environment and can correlate with pathogen prevalence, with lobsters in more diverse microbial states exhibiting lower infection prevalence than those with reduced diversity, highlighting potential links between microbial ecology and host health outcomes (Holt et al., 2019a). Despite its economic importance, *H. gammarus* remains comparatively understudied in terms of disease ecology, with only a limited but taxonomically diverse set of pathogens and parasites documented to date (Davies and Wootton, 2018). This may reflect true differences in exposures regimes and environmental conditions, or simply reduced research focus. Given the species’ narrower thermal tolerance and slower recovery rates, future climate stress could increase disease susceptibility. However, without long-term monitoring and mechanistic studies, predictive capacity remains limited.

In *J. edwardsii*, documented disease cases include necrotic shell conditions and parasitic infections, though large-scale disease-driven mortality events remain less prominent in literature (Zha et al., 2017, 2018). However, given the species’ prolonged larval phase and sensitivity to environmental variability, early life stage stress may have significant downstream impacts on health and recruitment success. Environmental stressors such as warming and contaminant exposure may alter both host immune function and microbial community composition, creating conditions that favor disease progression (Kim et al., 2025). Microbiome research in *J. edwardsii* and analysis of bacterial communities on tail fan necrosis lesions reveal that disease-associated cuticle microbiomes differ in composition, richness and variability from those of healthy tissues, suggesting that opportunistic microbial colonization and dysbiosis play roles in disease expression (Zha et al., 2019). Disease research in *J. edwardsii* is similarly sparse relative to climate change. While bacterial infections and parasitic organisms have been identified, large-scale disease-driven mortality events are not as prominently documented as in *H. americanus* populations. However, there have been documented cases of paralytic shellfish toxins (PST) reported in *J. edwardsii* along the coast of Tasmania due to toxic blooms of dinoflagellate *Alexandrium catenella* (Turnbull et al., 2020). Experimental studies show that toxic prey can transfer paralytic shellfish toxins to the hepatopancreas, and exposure can induce measurable stress-related physiological indicators even if gross health effects are limited (Turnbull et al., 2021; Madigan et al., 2018). When lobsters are fed a diet of exposed mussels, this toxin accumulates significantly (~ 14%) in the hepatopancreas (Turnbull et al., 2020). Exposed lobsters increased their feeding in correlation to toxin concentrations, demonstrating that growth and nutritional status are not reduced and lobster catchability is not affected (Turnbull et al., 2020). Infection by the oomycete *Haliphthoros* sp. caused significant mortality in pueruli and juvenile *J. edwardsii* particularly in individuals smaller than 30mm in carapace length (Diggles, 2001). The disease resulted in gill lesions, tissue damage, lethargy, reduced feeding and death, often occurring before or during moulting, while larger lobsters appeared more resistant to the infection (Diggles, 2001). However, given this species’ prolonged larval phase and climate sensitivity in recruitment dynamics, physiological stress at early life stages could have significant downstream population effects. The absence of documented large-scale disease outbreaks should not be interpreted as an absence of risk, but rather as a potential research gap.

Overall, available evidence suggests that disease in lobsters is best understood as an integrative outcome of cumulative environmental stress rather than the consequence of single pathogens alone. Climate change and contaminant exposure can interact to alter physiological stability, impair immune function and disrupt host-microbiome dynamics thereby increasing disease susceptibility. While *H. americanus* provides the clearest example of these multi-stressor interactions, limited disease and microbiome research likely reflect knowledge gaps rather than reduced vulnerability. Consequently, future research should adopt integrated, multi-stressor frameworks that link climate, toxicology, immunity and microbial ecology across species and life stages. Key disease agents and associated impacts are compared in **Table 3**, while **Figure 5** provides a conceptual overview of the interactions among environmental stressors, host responses and disease outcomes in lobsters.

**Table 3:** Disease and health challenges in lobster species.

Species	Disease type	Trigger/environmental link	Key effects	References
*H. americanus*	Epizootic Shell Disease (Bacterial)	Elevated temperature, low pH, contaminant exposure	Microbial dysbiosis, ↓ cuticle integrity, ↑ physiological stress, altered hemocytes responses	(Ishaq et al., 2023; Kim et al., 2025; Meres et al., 2012; McLean et al., 2018; Leblanc and Prince, 2012)
*H. americanus*	Gaffkemia (*Aerococcus viridans*)	Handling stress, high density conditions	Systemic bacterial infection, impaired immune function, increased mortality	(Clark et al., 2016; Stewart et al., 2004)
*H. gammarus*	Shell Disease (Bacterial)	Environmental stress, reduced water quality	Cuticle degradation, ↓ shell integrity, increased susceptibility to secondary stressors	(Davies et al., 2014a; Rowley and Coates, 2023)
*H. gammarus*	Unicellular pathogens	Environmental variability	↓ energy, ↑ mortality, ↓ growth	(Holt et al., 2018; Holt et al., 2019b)
*J. edwardsii*	Necrotic Shell Condition (Microbial dysbiosis)	Temperature stress, habitat conditions	Microbial dysbiosis, cuticle degradation, ↓ shell integrity, potential reductions in growth	(Zha et al., 2017; Zha et al., 2018; Zha et al., 2019)

**Figure 5:**
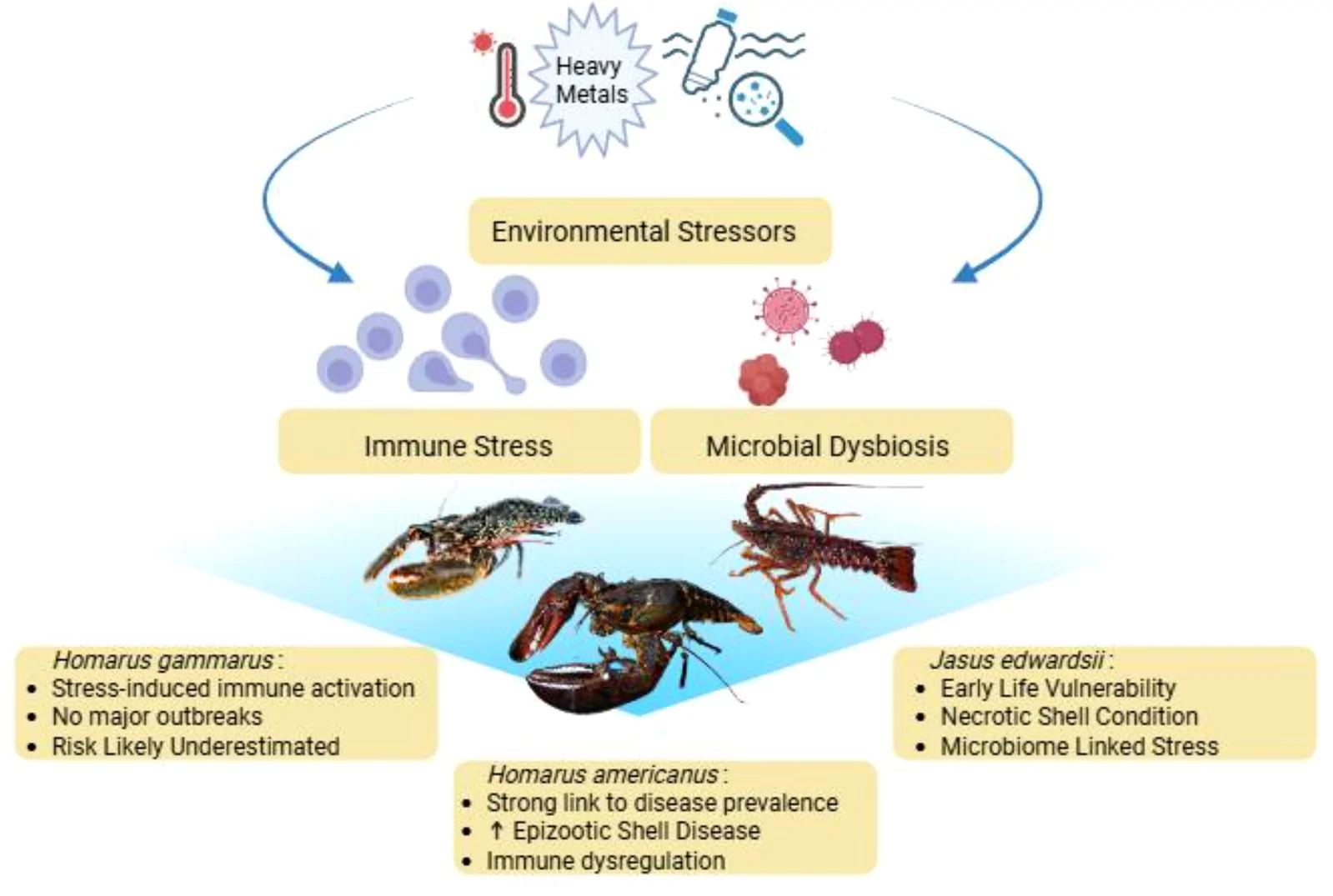
Pathways linking environmental stressors to physiological and health outcomes in lobsters. Environmental stressors such as rising temperatures, contaminants, and pathogens, can disrupt physiological homeostasis in marine crustaceans. These stressors may lead to immune system activation and dysregulation, as well as alterations in the composition and function of microbial communities. Together, these effects can increase susceptibility to disease and reduce overall fitness.

### 5.3 Knowledge gaps and future directions

Despite the economic and ecological importance of lobsters, substantial gaps remain in understanding how multiple stressors interact across species, life stages and environmental contexts. A key limitation in the current literature is the disproportionate focus on *Homarus americanus*, which, while well justified by its economic value, restricts broader comparative and predictive understanding across lobster taxa. In contrast, *H. gammarus* and *J. edwardsii* remain comparatively underrepresented, particularly in long-term monitoring and experimental studies addressing toxicology, disease dynamics and early life stage responses to environmental stressors.

Most existing research continues to examine climate stressors, contaminants, and disease in isolation, despite increasing evidence that these drivers interact to shape physiological performance and health outcomes. For example, contaminant exposure has been linked to oxidative stress biomarkers, while thermal stress independently activates a broader cellular stress response in the same species (see Section 4.2); whether these responses interact or compound one another remains unclear, illustrating a concrete gap in multi-stressor research on lobsters. Ocean acidification-disease interactions represent another underexplored axis of multi-stressor vulnerability: elevated CO_2_ alone has been shown to increase shell disease prevalence and reduce growth in early benthic-phase *H. americanus* (McLean et al., 2018), paralleling the thermal-oxidative stress overlap discussed above and reinforcing the need for experimental designs that test stressors in combination rather than isolation. Key gaps remain in multi-stressor experiments and cross-species frameworks to improve predictions of lobster resilience under accelerating environmental change. Future work should therefore prioritize studies that combine realistic environmental variability with comparative species approaches to strengthen predictive capacity across taxa.

## 6 Conclusion

This comparative review highlights both shared vulnerabilities and species-specific responses to climate change, contaminant exposure and disease across three ecologically and economically important lobster taxa. While ocean warming, ocean acidification and pollution consistently influence physiological function, outcomes are strongly mediated by differences in thermal tolerance, life-history traits, and habitat.

The American lobster currently provides the most well-documented example of climate-linked redistribution, contaminant accumulation and disease emergence, particularly in Southern New England, whereas comparable processes in *H. gammarus* and *J. edwardsii* remain less resolved, likely reflecting research and monitoring biases rather than true ecological resilience. As marine ecosystems continue to warm and anthropogenic pressures intensify, there is a clear need for integrative frameworks that explicitly connect physiology, toxicology, disease ecology, and fisheries management to better predict population-level responses under environmental change.
